# An assessment of terminology for intraspecific diversity in fishes, with a focus on “ecotypes” and “life histories”

**DOI:** 10.1002/ece3.7884

**Published:** 2021-07-13

**Authors:** Benjamin J. Clemens, Carl B. Schreck

**Affiliations:** ^1^ Oregon Department of Fish and Wildlife Corvallis OR USA; ^2^ Department of Fisheries and Wildlife Oregon State University Corvallis OR USA

## Abstract

Understanding and preserving intraspecific diversity (ISD) is important for species conservation. However, ISD units do not have taxonomic standards and are not universally recognized. The terminology used to describe ISD is varied and often used ambiguously. We compared definitions of terms used to describe ISD with use in recent studies of three fish taxa: sticklebacks (Gasterosteidae), Pacific salmon and trout (*Oncorhynchus* spp., “PST”), and lampreys (Petromyzontiformes). Life history describes the *phenotypic* responses of organisms to environments and includes biological parameters that affect population growth or decline. Life‐history pathway(s) are the result of different organismal routes of development that can result in different life histories. These terms can be used to describe recognizable life‐history traits. Life history is generally used in organismal‐ and ecology‐based journals. The terms paired species/species pairs have been used to describe two different phenotypes, whereas in some species and situations a continuum of phenotypes may be expressed. Our review revealed overlapping definitions for race and subspecies, and subspecies and ecotypes. Ecotypes are *genotypic* adaptations to particular environments, and this term is often used in genetic‐ and evolution‐based journals. “Satellite species” is used for situations in which a parasitic lamprey yields two or more derived, nonparasitic lamprey species. Designatable Units, Evolutionary Significant Units (ESUs), and Distinct Population Segments (DPS) are used by some governments to classify ISD of vertebrate species within distinct and evolutionary significant criteria. In situations where the genetic or life‐history components of ISD are not well understood, a conservative approach would be to call them phenotypes.

## Graphical abstract

The terminology used to describe intraspecific diversity is varied and often used ambiguously. “Ecotype” was originally used to describe patterns in genes and ecology, and recent studies employing this term tend to report a genetic basis in ISD. By contrast, “life history” describes biological parameters that affect demography, and this term tends to be used in organismal‐ and ecology‐based journals.

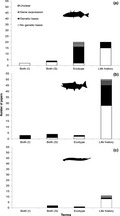

## 1 INTRODUCTION

*It is incumbent on any scientist, no matter the field of inquiry, to adhere to (or at least specify) definitions*. (Patten, 2015).

Intraspecific diversity (ISD) represents the “evolutionary potential within a species” (Haig et al., 2006), and understanding and preserving this diversity is an important conservation goal (COSEWIC, 2021; Ford, 2004; Waples, 2006). However, with some exceptions at the federal level (e.g., Designatable Units in Canada; COSEWIC, 2021) and Distinct Population Segments in the United States (Waples, 2006), units of ISD do not have taxonomic standards, are not universally recognized, and thus are contested (Ginsburg, 1937; Haig et al., 2006; Hubbs, 1943; Patten, 2015). Intraspecific diversity can be challenging to understand, given the variable roles of phenotype, genotype, and phenotypic plasticity (interactions of the genotype with the environment): Variability_Phenotype_ = Variability_Genotype_ + Variability_Environment_ + Variability_Genotype × Environment_ (DeWitt & Scheiner, 2004). Some researchers assess phenotypic expression relative to genotype and particular environments. However, an easier and therefore more common strategy among researchers is to focus on components of this relationship. Given these challenges, it is perhaps not surprising that the terminology for describing ISD is often used ambiguously. The ambiguous use of terminology to describe ISD ironically creates another impediment to understanding and thus preserving this diversity.

The scientific literature includes a plethora of terms to denote ISD. These include morphotypes (Chavarie et al., 2013; Collyer et al., 2015; Lessios & Weinberg, 1994); ecotypes (Arostegui et al., 2018; Cruz‐Font et al., 2019; Gregor, 1944) species pairs (Taylor, 1999); ecomorphotypes (Baker et al., 1998; Kloh et al., 2019; Segura‐Trujillo et al., 2018); ecophenotypes (Proćków et al., 2018; Schönborn & Peschke, 1988; Sorensen & Lindberg, 1991); polymorphisms (Jamie & Meier, 2020; Skúlason et al., 2019; Skúlason & Smith, 1995); and life histories (Stearns, 1989; Winemiller & Rose, 1992). Several of these terms have common roots and are often used interchangeably or in combination (e.g., Baker et al., 1998; Brannon et al., 2004; Chavarie et al., 2013; Palacios et al., 2012; Wood et al., 2008). The use of these terms may suggest perceived or actual novelty, a unique take on biological phenomena or an attempt to follow precedents of other published works. Although a diverse terminology can be useful in describing the existing diversity of evolved or expressed phenotypes, careful use of terms could improve knowledge transfer and clarity of understanding among scientists, policy makers, and fisheries managers. Here, we assess the use of terms to describe ISD in the peer‐reviewed scientific literature. We focused on two ISD terms that we believe have been used inconsistently and interchangeably—life histories and ecotypes.

Our goals were to assess the terminology for ISD and make recommendations for future use of these terms. Our four objectives were to (1) define key terms for intraspecies diversity using classical and authoritative sources that set a precedent and articulate clear definitions; (2) provide a meta‐review of evolution, traits, and ISD; (3) analyze trends over the last three decades (1990–2019) in the use of the terms “life history” and “ecotype” in the peer‐reviewed literature; and (4) compare the authoritative definitions with the trends in use of life history and ecotypes and make recommendations on future term use. For objectives 2 and 3, we focused on three fish taxa, including sticklebacks (Gasterosteidae), Pacific salmon and trout (*Oncorhynchus* spp., herein, “PST”), and lampreys (Petromyzontiformes) that represent a rich history of classical ecology and evolutionary studies (Bell & Foster, 1994; Docker, 2015, 2019; Groot & Margolis, 1991; Hardisty, 2006; Hendry et al., 2013; Hendry & Stearns, 2004; Orlov & Beamish, 2016a, b; Quinn, 2005; Wootton, 2009).

## 2 METHODS

### 2.1 Objective 1: Definitions

We found early uses of the terms ecotypes and life history in the literature. In papers that make a distinction across various animal taxa, we focused on information provided for fishes, for example, as by Haig et al. (2006). Our literature search included locating the first use of the term “ecotype” in the early 1900s and key publications by Stearns (1989, 1992). In addition, we found definitions for other terms that have been used synonymously with ecotype and life history.

### 2.2 Objective 2: Meta‐review of fish taxa

We conducted broad and succinct reviews of sticklebacks, PST, and lampreys that focused on books, book chapters, review articles, and other peer‐reviewed literature to provide and describe the number of species, their evolution, trait diversity, and use of terms to describe ISD. We chose to do a meta‐literature review because the exponential increase in articles for these species (e.g., Wootton, 2009) rendered exhaustive reviews untenable for the scope of this paper.

### 2.3 Objective 3: Trends in use of “life history” and “ecotype”

We conducted three independent searches for the use of the terms, “ecotypes” and “life history” for sticklebacks, PST, and lampreys using the advanced search option in the Web of Science search engine for articles in English, over 30 years (for years 1990–2019). Each search included the words “stickleback” or “*Oncorhyhnchus*” or “lamprey,” with at least one of the terms “ecotype life history” in the title of the article, using the operators: (TI=(ecotype OR life history) AND TI=(stickleback)). The same was done for “*Oncorhynchus*” and “lamprey.” These searches were executed between November 2020 and February 2021. Each article was reviewed to determine the focal species and phenotypes assessed; whether a genetic basis was identified for the diversity in phenotypes; and whether an article used both terms (ecotypes and life history) synonymously or both, but independently or used only one of the terms. Finally, the frequency of term use was calculated and compared among papers.

### 2.4 Objective 4: Compare definitions with term use and make recommendations

We compared definitions (Objective 1) with the meta‐review (Objective 2) and trends in use of the terms ecotypes and life history (Objective 3). We addressed the questions: Are there clear patterns in how terms are used in particular contexts? Do redundancies or ambiguities exist in the use of some terms that suggest that some terms ought not to be used?

## 3 RESULTS AND DISCUSSION

### 3.1 Objective 1: Definitions

In our search for definitions of ecotype and life history, it became apparent that several terms are used more‐or‐less synonymously (e.g., “species pairs”, “ecotypes”, and “life histories” in Taylor, 1999 and “races”, “phenotype”, “types”, and “subspecies” in Brannon et al., 2004). This entanglement of phenotypic terms was noted over eight decades ago: “The questions of what is a species, or a subspecies, or a race, or any classificatory category of specific or lower rank, cannot be disassociated from one another” (Ginsburg, 1937).

We compiled definitions of common terms used to describe ISD (Table 1). Generic terms used to describe ISD include “form” and “type.” Life history describes the *phenotypic* responses of organisms to environments and includes biological parameters that affect population growth and decline, including birth, survival, reproductive timing, reproductive investment, and mortality. Life‐history pathway(s) are the result of different developmental routes by an organism that are contingent upon the physiological status and genetic thresholds of that organism. The different developmental routes can result in different life histories. The terms paired species/species pairs have been used to describe two different phenotypes such as benthic versus limnetic sticklebacks or freshwater resident kokanee versus anadromous sockeye salmon (*O. nerka*; Taylor, 1999) and freshwater resident, nonfeeding brook lampreys versus anadromous and parasitic lampreys (Docker, 2009; Docker & Potter, 2019; Salewski, 2003). However, for lampreys, the more appropriate term would be “satellite species” and *not* “species pairs” (see below). Species pairs implies two phenotypes, whereas in some species and situations a continuum of phenotypes may be expressed. Our review revealed the ambiguity of the term, race, and the overlap in definitions of this term with subspecies. Classification of subspecies is controversial among taxonomists (Haig et al., 2006; Patten, 2015; de Queiroz, 2020), and a commonly accepted definition of subspecies remains elusive (Haig et al., 2006). Nevertheless, subspecies have recently been defined as components of a species that are incompletely speciated (Patten, 2015; de Queiroz, 2020; Table 1). We also found an overlap in definitions between subspecies and ecotypes. Ecotype was originally used to describe patterns in traits (genes) and ecology in the early 1900s (Gregor, 1944; Turesson, 1922). In essence, ecotypes are *genotypic* adaptations to particular environments. “Satellite species” is used for situations in which a parasitic lamprey yields two or more derived, nonparasitic lamprey species (Docker, 2009; Salewski, 2003; Vladykov & Kott, 1979). In some situations, these closely related lamprey species may not be distinct species (Docker, 2009). Designatable Units, Evolutionary Significant Units (ESUs), and Distinct Population Segments (DPS) are used by the Canadian and United States of America to classify ISD of vertebrate species along with distinct and evolutionary significant criteria. Evolutionary Significant Units are a special case of DPSs that have been used for PST (Table 1).

**TABLE 1:** Terms used to define diverse phenotypes of plants and animals, and the processes driving within‐species diversification. The terms are generally arranged from top to bottom by simple adaptive bifurcation to adaptive radiation. *Italicized* terms (“life history” and “ecotype”) are the focus of the present paper

Term	Definition	Process	Notes
Form	Term denoting a unique phenotype.	‐	Sometimes used in conjunction with other terms.
Type	Term denoting a unique phenotype.	‐	Sometimes used in conjunction with other terms (e.g., “life‐history type”).
*Life history*	Phenotypes of the same or similar species differing with respect to various life‐history parameters that are interrelated by trade‐offs among these parameters (Stearns, 1989).	?	The life‐history parameters include, among others, birth, size, growth characteristics, age and size at maturity, fecundity, offspring size and sex ratio, reproductive investments relative to age and size, mortality relative to age and size, and duration of life (Stearns, 1992).
	Reproductive effort related to age or life stage, and in response to factors that influence fecundity and survival. Thus, life histories reflect the expression of fitness‐related traits, including the timing and expression of the number, size, and life span of offspring, and size and age at maturity (Hutchings, 2004).	?	
*Life‐history pathway*	Alternative pathways of development that yield different life‐history traits, depending on the physiological status and genetic thresholds of a species. This can result in a diversity of life histories rather than a particular life history (Thorpe et al., 1998).	Phenotypic plasticity	
Paired species/species pair	Two phenotypes of the same species that differ in morphology, behavior, genetics, and ecology (Taylor, 1999).	Incipient ecological speciation	Speciation may occur at different rates in different locations and for different species, along a continuum of states (Hendry, 2009).
Races	“The terms ‘race’ and ‘subspecies’ are often used interchangeably” (Ginsburg, 1937).	?	
	Roughly equivalent to subspecies (Haig et al., 2006).	?	
Subspecies	“Heritable geographic variation in phenotype” (Patten, 2015).	?	Although species may go through a subspecies stage, all subspecies may not become species (Patten, 2015).
	“…subspecies are simply incompletely separated species within a more inclusive species” (de Queiroz, 2020).	?	
	“Unlike races, subspecies are animal kinds which are sufficiently clear‐cut as to be thought worthy of a place in the nomenclatorial system, but which do not give evidence of being completely differentiated”; “Incompleteness versus completeness of differentiation is the main test by which subspecies may be distinguished from species…”; “…subspecies in fishes are being shown to be ecological (or microgeographical) forms, which occupy diverse habitats in the same or in very broadly overlapping areas” (Hubbs, 1943).	?	Similar to “ecotype.”
*Ecotype*	Term originating from botany (e.g., Turesson, 1922 and Gregor, 1944). Mayr (1947) stated that “Ecotypes are populations or groups of populations and so are subspecies.”	?	
	“Distinct genotypes (or populations) within a species, resulting from adaptation to local environmental conditions; capable of interbreeding with other ecotypes or epitypes of the same species” (Hufford & Mazer, 2003).	?	
	“The term ecotype is proposed here as ecological unit to cover the product arising as a result of the genotypical response of an ecospecies to a particular habitat. The ecotypes are then the ecological subunits of the ecospecies, while the genotypes are purely Mendelian subunits of the genospecies. Knowledge of the ecology of an ecospecies presupposes knowledge of its most important ecotypes, and the knowledge of the ecology of the latter involves primarily a study of the variation and the distribution in nature of each of these ecotypes” (Turesson, 1922).	?	
Satellite species	Describes situations in which a single parasitic lamprey species gives rise to one *or more* nonparasitic species (Vladykov & Kott, 1979).	Incipient ecological speciation to speciation (Docker, 2009)	In some situations, these closely related lamprey species may not be distinct species (Docker, 2009).
Disegnatable Units	“Designatable units should be discrete and evolutionarily significant units of the taxonomic species, where ‘significant’ means that the unit is important to the evolutionary legacy of the species as a whole and if lost would likely not be replaced through natural dispersion.”	Human constructs and speciation	COSEWIC (2021)
Distinct Population Segment (DPS)	Any subspecies or distinct population segment of vertebrate species that interbreeds is reproductively isolated and is an evolutionarily significant unit (i.e., it provides a significant contribution to the genetic and ecological diversity) of the species (ESA, 1973; Waples, 2006).	Human constructs and speciation	
Evolutionary Significant Unit	Special case of DPS for Pacific salmon (*Oncorhynchus*). Legally listable entity used by the National Marine Fisheries Service and the U.S. Fish and Wildlife Service, under the U.S. Endangered Species Act (1973), to include distinct vertebrate populations with unique genetic diversity (Waples, 1991, 1995, 2006).	Human constructs and speciation	Based largely on Pacific salmon
	Context‐dependent population or population group that is arbitrarily chosen based on biological components (reproductively isolated, displays unique genetic, phenotypic, and ecological components), and economic, cultural, and social considerations (reviewed in Ford, 2004).		

### 3.2 Objective 2: Meta‐review of fish taxa

#### 3.2.1 Sticklebacks

Research on sticklebacks has focused primarily on one species, the threespine stickleback, *Gasterosteus aculeatus*, with fewer studies on ninespine stickleback, *Pungitius pungitius* (e.g., Table 2). The threespine stickleback has been a model organism for studying behavior, host‐parasite relationships, morphology, evolutionary ecology, and speciation (e.g., Baker et al., 2008; Bell & Foster, 1994; Hendry et al., 2009, 2013; McKinnon & Rundle, 2002; Schluter, 2010; Wootton, 2009). The overall trend with studies on the threespine stickleback has been the identification of numerous species, followed by lumping into one species, followed by a return to splitting the phenotypes back out into individual species in some geographical areas.

**TABLE 2:** Papers identified through Web of Science search (see text for details). The papers are arranged by sticklebacks, then *Oncorhynchus* spp., and then lampreys. Within each of these three taxa, the papers are organized by year of publication, and then alphabetically, by the authors’ last names. For terms, “1” = “life history” or “life histories”; “2” = “ecotype(s)”; “3” = both terms 1 and 2 were used synonymously; and “4” = both terms 1 and 2 were used independently (i.e., not synonymously)

Species	Phenotypes	Evidence for genetic basis	Terms	Reference
Threespine stickleback, *Gasterosteus aculeatus*	Age and body size at maturity, reproductive effort, and fecundity of ocean and lake phenotypes	No	4	Baker et al. (2019)
Ninespine stickleback, *Pungitius pungitius*	Body size at spawning, fecundity, and egg sizes of lake and stream phenotypes	No	1	Heins (2019)
Threespine stickleback, *G. aculeatus*	Melanophore expression, courtship rates, and parental care of white and normal male phenotypes	Unclear	2	Haley et al. (2019)
Threespine stickleback, *G. aculeatus*	Genotyping and gene expression in lake and stream phenotypes	Yes	2	Huang et al. (2019)
Threespine stickleback, *G. aculeatus*	Differences in major histocompatibility complexes and assortative mating of lake and river phenotypes	Yes	2	Gahr et al. (2018)
Threespine stickleback, *G. aculeatus*	Gene expression to different osmotic environments between freshwater and marine phenotypes	Yes	2	Rastorguev et al. (2018)
Threespine stickleback, *G. aculeatus*	Gene expression between marine and freshwater phenotypes exposed to different salinities	Not per se; however, different gene *expression*	2	Gibbons et al. (2017)
Threespine stickleback, *G. aculeatus*	Gene expression of quantitative trait loci between marine and freshwater (stream) phenotypes, incl. exposure to different salinities	Yes	2	Ishikawa et al. (2017)
Threespine stickleback, *G. aculeatus*	Growth rates, parasitic infection rates, variation in major histocompatibility complexes, and survival of lake and river phenotypes	Yes	2	Kaufman et al. (2017)
Threespine stickleback, *G. aculeatus*	Reaction norms of body size, coloration, and transcriptional responses to different temperatures	Yes	1	Kim et al. (2017)
Threespine stickleback, *G. aculeatus*	Osmoregulation differences between anadromous and stream phenotypes	Yes	2	Kusakabe et al. (2017)
Threespine stickleback, *G. aculeatus*	Bimodality in age and size at spawning	No	1	Rollins et al. (2017)
Threespine stickleback, *G. aculeatus*	Physiology and gene expression upon exposure to winter temperatures in marine and freshwater phenotypes	Not per se; however, different gene *expression*	2	Gibbons et al. (2016)
Threespine stickleback, *G. aculeatus*	Genetic and morphology of lake and stream phenotypes	Yes	2	Hanson et al. (2016)
Threespine stickleback, *G. aculeatus*	Changes in body size, clutch mass, fecundity, egg mass following predator introduction	No	1	Heins et al. (2016)
Threespine stickleback, *G. aculeatus*	Body size and age at spawning, fecundity, clutch mass, and egg mass following introduction of anadromous stickleback into lakes	No	1	Kurz et al. (2016)
Threespine stickleback, *G. aculeatus*	Genotyping of marine and freshwater phenotypes	Yes	2	Liu et al. (2016)
Threespine stickleback, *G. aculeatus*	Micro RNA regulatory activity of marine and freshwater phenotypes	Yes	2	Rastorguev et al. (2016)
Threespine stickleback, *G. aculeatus*	Morphology of lake and stream phenotypes	Yes	2	Lucek et al. (2014)
Threespine stickleback, *G. aculeatus*	Transgenerational effects of CO_2_ on parental fecundity, offspring survival, growth, and otolith attributes	No	1	Schade et al. (2014)
Threespine stickleback, *G. aculeatus*	Different immune responses and susceptibility of lake and river phenotypes to an eye fluke	Ecotypes genetically different, but no genetic evidence for pathological responses	2	Scharsack and Kalbe (2014)
Threespine stickleback, *G. aculeatus*	Reproductive effort and fecundity among phenotypes from different environments	Yes	1	Baker et al. (2013)
Threespine stickleback, *G. aculeatus*	Body size and age at spawning, fecundity, egg mass, and reproductive effort of anadromous and freshwater phenotypes	Yes	3	Karve et al. (2013)
Threespine stickleback, *G. aculeatus*	Thyroid concentrations in marine and stream phenotypes	No	3	Kitano and Lema (2013)
Threespine stickleback, *G. aculeatus*	Gene expression in response to infection of lake and stream phenotypes	Not per se; however, different gene *expression*	2	Lenz et al. (2013)
Threespine stickleback, *G. aculeatus*	Fecundity, egg size, and morphology of marine and lake phenotypes	No	4	Oravec and Reimchen (2013)
Threespine stickleback, *G. aculeatus*	Different trophic ecology and mercury accumulation of limnetic and benthic phenotypes	No	2	Willacker et al. (2013)
Threespine stickleback, *G. aculeatus*	Body size, fecundity, and spawn timing of lake and stream phenotypes	Yes	1	Moser et al. (2012)
Threespine stickleback, *G. aculeatus*	Rearing marine phenotype in different habitats results in the derivation of benthic and limnetic phenotypes	No	3	Wund et al. (2012)
Threespine stickleback, *G. aculeatus*	Egg size, fecundity, body size, and age	Not directly, but inferred	1	Baker et al. (2011)
Threespine stickleback, *G. aculeatus*	Body morphology and gill raker number of lake and stream phenotypes	Yes	2	Berner et al. (2011)
Threespine stickleback, *G. aculeatus*	Diversity in major histocompatibility complexes between lake and stream phenotypes	Yes	2	Eizaguirre et al. (2011)
Ninespine stickleback, *P. pungitius*	Otolith microchemistry of brackish and anadromous phenotypes	No	1	Arai et al. (2010)
Threespine stickleback, *G. aculeatus*	Age, length, weight, sex ratio, spawn timing, fecundity	No	1	Patimar et al. (2010)
Threespine stickleback, *G. aculeatus*	Assortative mating behaviors of lake, stream, and hybrid phenotypes	Yes	2	Raeymaekers et al. (2010)
Threespine stickleback, *G. aculeatus*	Reproductive effort, fecundity, egg size, age, and body size at spawning of marine (anadromous and strictly marine) and freshwater (lake and stream) phenotypes	No	1	Baker et al. (2008)
Threespine stickleback, *G. aculeatus*	Immune responses of lake and stream phenotypes	No	2	Scharsack et al. (2007)
Ninespine sticklebacks, *Pungitius* spp.	Otolith microchemistry of brackish, anadromous, and freshwater phenotypes	No	1	Arai and Goto (2005)
Threespine stickleback, *G. aculeatus*	Reproductive allocation of benthic and limnetic phenotypes	No	3	Baker et al. (2005)
Threespine stickleback, *G. aculeatus*	Body size, fecundity, egg size, and relative clutch mass	No	1	Baker and Foster (2002)
Threespine stickleback, *G. aculeatus*	Body size, fecundity, egg size, and gonad mass among years	No	1	Poizat et al. (2002)
Threespine stickleback, *G. aculeatus*	Reproductive and hatch timing and age of offspring maturity of river phenotype	No	1	Saito and Nakano (1999)
Threespine stickleback, *G. aculeatus*	Body size, body morphology, reproductive effort, egg mass, egg size, and fecundity of anadromous, lake, and stream phenotypes	No	1	Baker et al. (1998)
Threespine stickleback, *G. aculeatus*	Body morphology and clutch volume of benthic and limnetic phenotypes	No	1	Foster et al. (1992)
Threespine stickleback, *G. aculeatus*	Growth rate differences between anadromous and stream phenotypes	Not directly, but inferred	1	Snyder (1991)
Threespine stickleback, *G. aculeatus*	Body size, reproductive timing, fecundity of anadromous and stream phenotypes	No	1	Snyder and Dingle (1990)
Rainbow trout, *O. mykiss*	Gene expression by sex in anadromous and resident phenotypes	Not per se; however, different gene *expression*	1	Hale et al. (2018)
Rainbow trout, *O. mykiss*	Marine trophic position, foraging area, somatic lipids, and stable isotopes of summer (stream maturing) and winter (ocean maturing) phenotypes	No	2	Lamperth et al. (2018)
Coho salmon, *O. kisutch*	Migration behavior	No	1	Faukner et al. (2017)
Sockeye salmon, *O. nerka*	Genetics of anadromous and resident phenotypes	Yes	1	Samarasin et al. (2017)
Chinook salmon, *O. tshawytscha*	Genetics of precocial jack and "normal" male phenotypes	Yes	1	Forest et al. (2016)
Sockeye salmon, *O. nerka*	Genotyping of anadromous and resident phenotypes	Yes	3	Nichols et al. (2016)
Rainbow trout, *O. mykiss*	Genetics and size at age of anadromous and resident phenotypes	Yes	1	Phillis et al. (2016)
Sockeye salmon, *O. nerka*	Genetics and body size at maturity, behavior, morphology, and gill raker number of anadromous and resident phenotypes	Yes	4	Moreira and Taylor (2015)
Chinook salmon, *O. tshawytscha*	Emigration date and body size of transplanted fish	No	1	Roddam and Ward (2015)
Rainbow trout, *O. mykiss*	Maturation rates relative to anadromous and resident phenotypes	Yes, inferred	1	Berejikian et al. (2014)
Coho salmon, *O. kisutch*	Growth rate, body size, freshwater residence, and emigration timing	No	1	Craig et al. (2014)
Coho salmon, *O. kisutch*	Habitat use, age, growth rate, freshwater residence timing, emigration timing, and survival	No	1	Jones et al. (2014)
Rainbow trout, *O. mykiss*	Influence of sex on anadromous vs. resident phenotypes	No	1	Ohms et al. (2014)
Rainbow trout, *O. mykiss*	Smoltification and maturation rates and somatic growth of anadromous and resident phenotypes	No	1	Sloat and Reeves (2014)
Rainbow trout, *O. mykiss*	Smoltification, residency, and growth rates of anadromous and resident phenotypes	No	1	Benjamin et al. (2013)
Rainbow trout, *O. mykiss*	Genotyping of anadromous and resident phenotypes	Yes	3	Hale et al. (2013)
Rainbow trout, *O. mykiss*	Gene flow between anadromous and resident phenotypes	No	1	Van Doornik et al. (2013)
*Oncorhynchus* spp.	Stray rates of different species, including ocean and stream phenotypes of Chinook salmon	No	1	Westley et al. (2013)
Chinook salmon, *O. tshawytscha*	Ocean migration paths of ocean and stream phenotypes	No	1	Sharma and Quinn (2012)
Rainbow trout, *O. mykiss*	Growth rate and timing and ages of anadromous and resident phenotypes	Yes, inferred	1	Sogard et al. (2012)
Rainbow trout, *O. mykiss*	Growth rates and ages of stream and lake phenotypes	No	1	Arismendi et al. (2011)
Rainbow trout, *O. mykiss*	Physiological profiles of anadromous and resident phenotypes	Not per se; however, different gene *expression*	1	Hanson et al. (2011)
Chinook salmon, *O. tshawytscha*	Heritability of size at age, and age at maturity	Yes	1	Kinnison et al. (2011)
Chinook salmon, *O. tshawytscha*	Natal origin and migration history	Yes	1	Miller et al. (2011)
Sockeye salmon, *O. nerka*	Genetics of anadromous and resident phenotypes	Yes	1	Waples et al. (2011)
Chinook salmon, *O. tshawytscha*	Genotyping and early life stage survival and growth	Yes	1	Evans et al. (2010)
Sockeye salmon, *O. nerka*	Otolith microchemistry of anadromous and resident phenotypes	No	2	Godbout et al. (2010)
Westslope cutthroat trout, *O. clarkii lewisi,* and rainbow trout, *O. mykiss*	Distribution, growth rate, and survival of two species	Yes	1	Rasmussen et al. (2010)
Coho salmon, *O. kisutch*	Growth rates, adult survival, body size at maturity, run timing, and relative reproductive success of hatchery fry and hatchery smolts	No	1	Thériault et al. (2010)
Rainbow trout, *O. mykiss*	Genetic diversity, effective population size of anadromous and resident phenotypes	Yes	1	Van Doornik et al. (2010)
Chinook salmon, *O. tshawytscha*	Body length and survival of fall parr, age‐0 smolts, and age‐1 smolts	No	1	Copeland and Venditti (2009)
Sockeye salmon, *O. nerka*	Growth rates, smolt sizes, ages of seaward migration and maturity, and ocean survival	No	1	Rich et al. (2009)
Chum salmon, *O. keta*	Emigration timing, growth rates, condition factor, and body size at ocean entry	No	1	Saito et al. (2009)
Sockeye salmon, *O. nerka*	Variable freshwater and ocean residence, ocean survival, maturity rates, and recruits per spawner	No	1	Lessard et al. (2008)
Rainbow trout, *O. mykiss*	Genotyping of anadromous and resident phenotypes	Yes	1	Narum et al. (2008)
Chinook salmon, *O. tshawytscha*	Emergence timing, growth rates, smolt rates (and timing), and maturation rates	No	1	Beckman et al. (2007)
*Oncorhynchus* spp.	Fry habitat, freshwater residency, ocean migration behavior, ocean duration, age at maturation, spawning location, spawning behavior and timing, and semelparity	No	1	Esteve and McLennan (2007)
Sockeye salmon, *O. nerka*	Genetics of anadromous and resident phenotypes	Yes	1	Pavey et al. (2007)
Coho salmon, *O. kisutch*	Age at maturity, size‐specific survival, and reproductive success	Unclear	1	Snover et al. (2006)
Sockeye salmon, *O. nerka*	Oxygen isotopes cannot be used to discern anadromous and resident life history in fossil bones	No	1	Zazzo et al. (2006)
Sockeye salmon, *O. nerka*	Freshwater longevity of jacks and older males	No	1	Carlson et al. (2004)
Masu salmon, *O. masou*	Growth rates, maturation timing of anadromous and resident phenotypes	No	1	Yamamoto (2004)
Sockeye salmon, *O. nerka*	Body size, sex ratio, age at maturity, and freshwater and ocean residence times by run timing	Yes	4	Fillatre et al. (2003)
Chinook salmon, *O. tshawytscha*	Genetic components of the jack male reproductive strategy	Yes	1	Heath et al. (2002)
Masu salmon, *O. masou*	Different male phenotypes (body sizes, behaviors) employ different mating tactics	No	1	Yamamoto and Edo (2002)
Sockeye salmon, *O. nerka*	Body sizes, age structure, and body depths of males	No	1	Hendry and Quinn (1997)
Rainbow trout, *O. mykiss*	Genetics of run timing	Yes	2	Nielsen and Fountain (1997)
Sockeye salmon, *O. nerka*	Morphological and genetic differences between stream‐ and beach‐spawning resident phenotypes	Yes	4	Taylor et al. (1997)
Chinook salmon, *O. tshawytscha*	Natural and hatchery origin, body size at age, and body size at maturity of ocean and stream phenotypes	Yes, inferred	1	Unwin and Glova (1997)
Sockeye salmon, *O. nerka*	Genetics of anadromous and resident phenotypes	Yes and No	3	Taylor et al. (1996)
Sockeye salmon, *O. nerka*	Egg size, body morphology, and spawning gravel size	No	1	Quinn et al. (1995)
Cutthroat trout, *O. clarki bouvieri*	Body size at age, migration strategy and timing, sex ratio, age of spawning	No	1	Gresswell et al. (1994)
Masu salmon, *O. masou*	Body size and age at maturity of anadromous and resident males	No	1	Tsiger et al. (1994)
Chinook salmon, *O. tshawytscha*	Age at maturity, body size at age, body mass, and timing of arrival on spawning grounds of ocean and stream phenotypes	No	1	Quinn and Unwin (1993)
Chinook salmon, *O. tshawytscha*	Growth rate and seawater adaptability of ocean and stream phenotypes	Yes	1	Clarke et al. (1992)
Chinook salmon, *O. tshawytscha*	Habitat preference, distribution, abundance, body size, migration and residence timing, and seawater tolerance of the ocean phenotype	No	1	Johnson et al. (1992)
Chum salmon, *O. keta*	Downstream migration, age at maturity, body size, egg size, body meristics, and morphology of early and late spawning phenotypes	No	3	Tallman and Healey (1991)
Chinook salmon, *O. tshawytscha*	Differences in the duration of stream residence, body size, agonistic behavior, and salinity tolerance among populations	Yes, inferred	1	Taylor (1990a)
Chinook salmon, *O. tshawytscha*	Duration of freshwater residence, distribution, migration distance, and growth rates of ocean and stream phenotypes	Yes	1	Taylor (1990b)
Chinook salmon, *O. tshawytscha*	Rheotaxis, aggression, and growth rates of ocean and stream phenotypes	Yes, inferred	1	Taylor (1990c)
Multiple lamprey species	Parasitic and nonparasitic phenotypes	No	1	Evans and Limburg (2019)
Pacific lamprey, *Entosphenus tridentatus*	Genotyping and body morphology and egg mass of ocean and stream maturing phenotypes	Yes	2	Parker et al. (2019)
European river lamprey, *Lampetra fluviatilis*, and European brook lamprey, *L. planeri*	Genotyping of freshwater nonparasitic, freshwater parasitic, and anadromous parasitic species	Yes	3	Hume et al. (2018)
European river lamprey, *L. fluviatilis*, and European brook lamprey, *L. planeri*	Genotyping of freshwater nonparasitic and anadromous parasitic species	Yes	3	Rougemont et al. (2017)
Least brook lamprey, *L. aepyptera*, and American brook lamprey, *Lethenteron appendix*	Strategies for lipid accumulation in two freshwater, nonparasitic species	No	1	Evans and Bauer (2016)
Chestnut lamprey, *Icthyomyzon castaneus*, and northern brook lamprey, *I. fossor*	Gene expression in freshwater parasitic and freshwater nonparasitic species	Not per se; however, different gene *expression*	1	Spice et al. (2012)
Pacific lamprey, *E. tridentatus*	Body morphology, fecundity, and physiology of ocean and stream maturing phenotypes	No	1	Clemens et al. (2013)
European river lamprey, *L. fluviatilis*, and European brook lamprey, *L. planeri*	Mating behavior of freshwater nonparasitic, freshwater parasitic, and anadromous parasitic species	No	1	Hume et al. (2013)
Arctic lamprey, *Lethenteron camtschaticum*	Genetics of anadromous parasitic and freshwater nonparasitic phenotypes	Yes	1	Yamazaki and Nagai (2013)
Sea lamprey, Petromyzon *marinus*	Length and weight at maturity, sex ratio, female gonad weight of anadromous parasitic species	No	1	Beaulaton et al. (2008)
Sea lamprey, P. *marinus,* and American brook lamprey, *L. appendix*	Physiology of transformation and sexual maturation in parasitic and nonparasitic species	Not per se; however, different gene *expression*	1	Youson et al. (2006)
Sea lamprey, P. *marinus*	Body size, growth rate, and age of larvae and transformers of landlocked parasitic species	No	1	Zerrenner and Marsden (2006)
Sea lamprey, P. *marinus*	Sex ratio, body size, and age at transformation of freshwater parasitic species	No	1	Zerrenner and Marsden (2005)
Western brook lamprey, *L. richardsoni*	Internal morphology and inferred physiology of parasitic and nonparasitic phenotypes	No	1	Youson and Beamish (1991)

In the early 1900s, taxonomists struggled with the wide phenotypic diversity of threespine stickleback and several phenotypes were initially believed to be separate species (Wootton, 2009). This diversity is captured in the following quote: “Race ranking may be accorded forms, like local types of *Gasterosteus aculeatus*, which are so confusingly numerous or so complex in characters, and so complicated in genetic and geographical relationship, as to transcend any ordinary scheme of zoological nomenclature” (Hubbs, 1943). It has since been argued that the threespine stickleback is a “raceme” (persistent lineage [marine phenotype] out of which multiple lineages [anadromous and freshwater phenotypes] diverge and quickly end in extinction) or “species complex,” composed of thousands of diverse populations that have evolved numerous times in particular locations (Bell & Foster, 1994; Hendry et al., 2013; Schluter & Conte, 2009; Wootton, 2009). Others refer to the diversity within threespine stickleback by calling the species a “superspecies” (Baker et al., 2008).

Stickleback speciation is complex and involves multiple traits. This speciation occurs rapidly in diverse geographical areas. Natural selection, sexual selection, standing genetic variation, mutation, and genetic recombination have led to rapid reproductive isolation and speciation that has occurred since the last glaciers ca. 9,000–13,000 years ago (Hendry et al., 2013; McKinnon & Rundle, 2002; Schluter, 2010; Schluter & Conte, 2009; Wootton, 2009). In the mid‐ to late‐1900s, research on sticklebacks examined the variation and adaptive significance of phenotypic traits including body shape and size, body armor (bony plates), spines and skeletal structure, spawning coloration, life‐history characteristics, and behavior. In the latter part of this period, research focused on the adaptive radiation and reproductive isolation of sticklebacks in lakes. In the 2000s, genomic studies on sticklebacks revealed insights into associations between phenotype, genotype, and selective factors (Wootton, 2009).

Phenotypic and genotypic differences in threespine stickleback have been found among marine, anadromous, freshwater resident populations (lakes and streams), and between phenotypes within these habitats (e.g., limnetic vs. benthic phenotypes/species; Table 2). This diversity has been identified as “species pairs” (Hendry et al., 2009; Taylor, 1999; Wootton, 2009), “ecomorph pairs” (Wootton, 2009), and “ecotypes” (Table 2; Hendry et al., 2013; Taylor, 1999). Life‐history diversity has also been examined (Table 2; Baker et al., 2008), and “life history” can be used to describe phenotypic characteristics in life‐history parameters across multiple lineages without having to demonstrate a genotypic association—unlike “ecotypes.” Some of these phenotypes of threespine stickleback show sufficient reproductive isolation and phenotypic and genotypic differences to warrant calling them separate species, although they still bear the same scientific name (Schluter, 2010; Wootton, 2009). For example, “limnetic” and “benthic” phenotypes/species have been shown to be adaptive in the littoral zone (benthic species/phenotype) and limnetic zone (limnetic species/phenotype) within some lakes (Schluter, 2010) and phenotypes associated with different lake substrates (lava vs. mud; Kristjánsson et al., 2002) in ways that reduce competition for resources (Schluter, 2010). In addition, some phenotypic and genotypic divergence in lakes has been attributed to predators and prey (Miller et al., 2019; Millet et al., 2013), and parasitism may also influence divergence leading to speciation between limnetic and benthic threespine sticklebacks (Schluter, 2010).

The appropriate terminology for describing threespine stickleback diversity may depend on the population(s) in question. This is because speciation within sticklebacks occurs along a continuum, from “continuous variation within panmictic populations” on one end to “complete and irreversible reproductive isolation” on the other, with factors affecting the divergence of populations along this continuum (Hendry et al., 2009). Hendry et al. (2009) reported that most stickleback populations are on the front end of this spectrum, “…even though some of these [populations] show evidence of disruptive selection and positive assortative mating.”

#### 3.2.2 Oncorhynchus

The genus *Oncorhynchus* includes five species of Pacific salmon and seven species of Pacific trout (Quinn, 2005; Penaluna et al., 2016). Pacific salmon and trout (PST) are iconic and important species culturally, economically, and recreationally (Lichatowich, 1999; Behnke, 2002; Penaluna et al., 2016). Research on PST has been important for informing biology and fisheries management (Groot & Margolis, 1991; Behnke, 2002; Penaluna et al., 2016), and ecology and evolutionary processes (Hendry & Stearns, 2004; Quinn, 2005; Stearns & Hendry, 2004; Waples & Hendry, 2008).

Modern PST are approximately 6–20 million years old, and further speciation and intraspecific diversification has been occurring ever since (Stearley & Smith, 1993; Montgomery, 2000; Waples et al., 2008; Penaluna et al., 2016). Significant geologic activity, including tectonic action, volcanism, and cycles of glaciation and de‐glaciation, occurred and thus has been implicated in influencing the speciation of PST (Montgomery, 2000; Penaluna et al., 2016). This geologic activity would have also resulted in creation of river drainages and thus geographical isolation that influenced PST speciation (Montgomery, 2000). Pacific salmon and trout exhibit a general pattern of isolation‐by‐distance, with populations near each other being more closely related than those further away (apart from sockeye salmon *O. nerka*; Waples et al., 2008; Wood et al., 2008). Pacific salmon and trout home to their natal streams and lakes, and this results in structured populations that are locally adapted to particular environments (Brannon et al., 2004; Hendry et al., 2004a; Quinn, 2005; Waples et al., 2001, 2008). Pacific salmon and trout have been described as “…different populations [that] represent ecological types referred to as spring‐, summer‐, fall and winter‐run segments, as well as stream‐ and ocean‐type, or stream‐ and ocean‐maturing life history forms” (Brannon et al., 2004).

Important diversification in PST occurs below the species level (Behnke, 2002). Traits of PST that diverge at the intraspecific level include run timing (Brannon et al., 2004; Groot & Margolis, 1991), anadromy/freshwater residency (Hendry et al., 2004b; Quinn & Myers, 2004), ocean residency, fecundity, territoriality, iteroparity/semelparity, and precocity versus larger and older spawning types (Table 2; see also Fleming & Reynolds, 2004; Quinn & Myers, 2004; Quinn, 2005). This ISD is a continuum determined by a suite of traits that are influenced along seasonal changes in environmental conditions (i.e., temporal clines). One key temporal cline is water temperature, which affects larval development, juvenile residence, and spawn timing (Brannon et al., 2004; Quinn & Myers, 2004; Waples et al., 2001). The diversity in life histories and genetics within PST exhibits a direct and strong correlation (Waples et al., 2001). In addition, life‐history traits in PST are directly related to evolutionary fitness and thus are subjected to strong and consistent selection (Carlson & Seamons, 2008; Hutchings, 2004). Nevertheless, many questions remain about the extent to which the ISD in PST is a result of phenotypic plasticity versus genetic adaptation (Hendry et al., 2004b; Waples et al., 2001; Waples & Hendry, 2008).

Several terms have been used to describe ISD in PST, including “morphotypes”, phenotypes, populations, stocks, “life history forms”, “life history types”, “ecological types”, “races”, “phenotype”, “forms”, “types”, and “subspecies” (Healey, 1991; Waples et al., 2001; Behnke, 2002; Brannon et al., 2004; Penaluna et al., 2016)—and this list is not exhaustive. The prevailing use of the term “life history” can be found in key tomes (e.g., Behnke, 2002; Groot & Margolis, 1991). Some authors combine use of terms such as “life history ecotypes” (Wood et al., 2008). In addition, some PST populations have received the designation of ESUs (Table 1). This designation enables tracking of demographic characteristics relative to population status.

#### 3.2.3 Lampreys

Lampreys are basal vertebrates (Docker et al., 2015; Janvier, 2008) that first appeared in the fossil record 360 million years ago (Gess et al., 2006)—long before teleost fishes like PST and sticklebacks appeared. Forty‐two to 45 species of lampreys exist (Maitland et al., 2015; Potter et al., 2015; Riva‐Rossi et al., 2020), including 2–26 species that are freshwater resident “brook” lampreys without a parasitic life stage, nine freshwater resident parasites, and 10 anadromous and parasitic species (Maitland et al., 2015; Riva‐Rossi et al., 2020).

Phenotypic diversity in lampreys has been characterized by the feeding (parasitic versus. nonfeeding) and migratory behavior (anadromous or resident; Salewski, 2003; Vladykov & Kott, 1979). The brook lampreys are relatively small in body size and females exhibit low fecundity, whereas the anadromous lampreys are relatively large and exhibit correspondingly higher fecundities (Docker, 2009; Docker & Potter, 2019; Salewski, 2003). The closely related pairs or groups of brook and anadromous lampreys have been termed “paired species” or “species pairs,” “satellite species” (more than two species), “life histories” (Docker, 2009; Docker & Potter, 2019; Salewski, 2003; Vladykov & Kott, 1979), and recently “ecotypes” (Docker & Potter, 2019; Rougemont et al., 2017). We argue that paired species/species pairs confuses ISD and interspecies diversity of lampreys with that of teleosts (e.g., Taylor, 1999); thus, these two terms should probably be avoided when discussing diversity in lampreys. By contrast, satellite species has a historical context (Vladykov & Kott, 1979) and makes sense because of the definition provided, which encompasses both ISD and *inter*species diversity (Table 1). Ecotypes are gaining in use for lampreys (Table 2), though it makes more sense to use this term in terms ISD and *not* for interspecies diversity. Life history could reasonably be used to describe recognizable differences in life‐history traits for ISD in lampreys. A review of studies on parasitic and nonparasitic species pairs of lampreys identified a continuum of genetic and phenotypic divergence within‐species pairs, with the term “ecotype” being used to indicate different phenotypic expression *and* partial or full reproductive isolation, whereas life history was used to indicate trade‐offs in body size and fecundity associated with feeding type (parasitic or nonfeeding) and anadromy versus freshwater residency (Docker & Potter, 2019).

The level of genetic relatedness between species pairs depends on the geographic location and circumstances. In some situations, closely related parasitic lamprey and nonparasitic brook lamprey can reproduce together; thus, they may more aptly be called phenotypes of the same species. Examples of this include the European river lamprey (*Lampetra fluviatilis*) and European brook lamprey (*L. planeri*; Rougemont et al., 2015), and the resident parasitic silver lamprey (*Ichthyomyzon unicuspis*), and nonparasitic northern brook lamprey (*I. fossor*; Docker et al., 2012). In other situations, these phenotypes exhibit discrete genetic differences, such as among specimens of parasitic western river lamprey (*L. ayresii*) and the closely related western brook lamprey (L. *richardsoni*) and other *Lampetra* species along the west coast of North America (Boguski et al., 2012), and among allopatric European river lamprey and European brook lamprey (Rougemont et al., 2017). These satellite species were originally identified as separate species (Docker, 2009; Vladykov & Kott, 1979). Resident brook lampreys are expected to display more population structure within a particular river basin than anadromous lampreys, as demonstrated for western brook lamprey (*L*. *richardsoni*; Spice et al., 2019). Anadromous lampreys do not home to their natal streams, and so they display less genetic stock structure (Bergstedt and Seelye., 1995; Bryan et al., 2005; Spice et al., 2012).

More recently, research into Pacific lamprey, *Entosphenus tridentatus*, has revealed another form of phenotypic diversity beyond feeding and migratory behavior: bimodal differences in maturation timing. Research into body morphology, gonadosomatic index (GSI), and maturation levels (determined by gonadal histology) revealed phenotypic differences in maturation timing, which were named “stream maturing” and “ocean maturing” Pacific lamprey (Clemens et al., 2013). It was hypothesized that the less‐mature life history or phenotype was the commonly recognized stream maturing phenotype that would be expected to spawn one or more years after entering freshwater, whereas the formerly unrecognized ocean maturing form (which is more sexually mature upon entering freshwater) might spawn within the same year of entering freshwater (Clemens et al., 2013). The ocean maturing phenotype was found in the Klamath River estuary (California, USA, at the river mouth, river kilometer 0). A separate study conducted at this same location verified the existence of stream maturing and ocean maturing ISD in Pacific lamprey, via single nucleotide polymorphism markers and GSI. This phenotypic diversity was initially referred to as “life histories” (Clemens et al., 2013) and then more recently as “ecotypes” (Parker et al., 2019).

In summary, closely related parasitic and nonparasitic lampreys have been called paired species, species pairs, satellite species, life histories, and ecotypes. Stream maturing and ocean maturing phenotypes of Pacific lamprey have been called life histories and ecotypes. We argue that paired species/species pairs should not be used to describe ISD or interspecies diversity in lampreys. By contrast, satellite species encompasses both ISD *and* interspecies diversity. Ecotypes should be used in terms of ISD and *not* for interspecies diversity. Life history could reasonably be used to describe recognizable differences in life‐history traits for ISD in lampreys.

### 3.3 Objective 3: Trends in use of “life history” and “ecotype”

**FIGURE 1:**
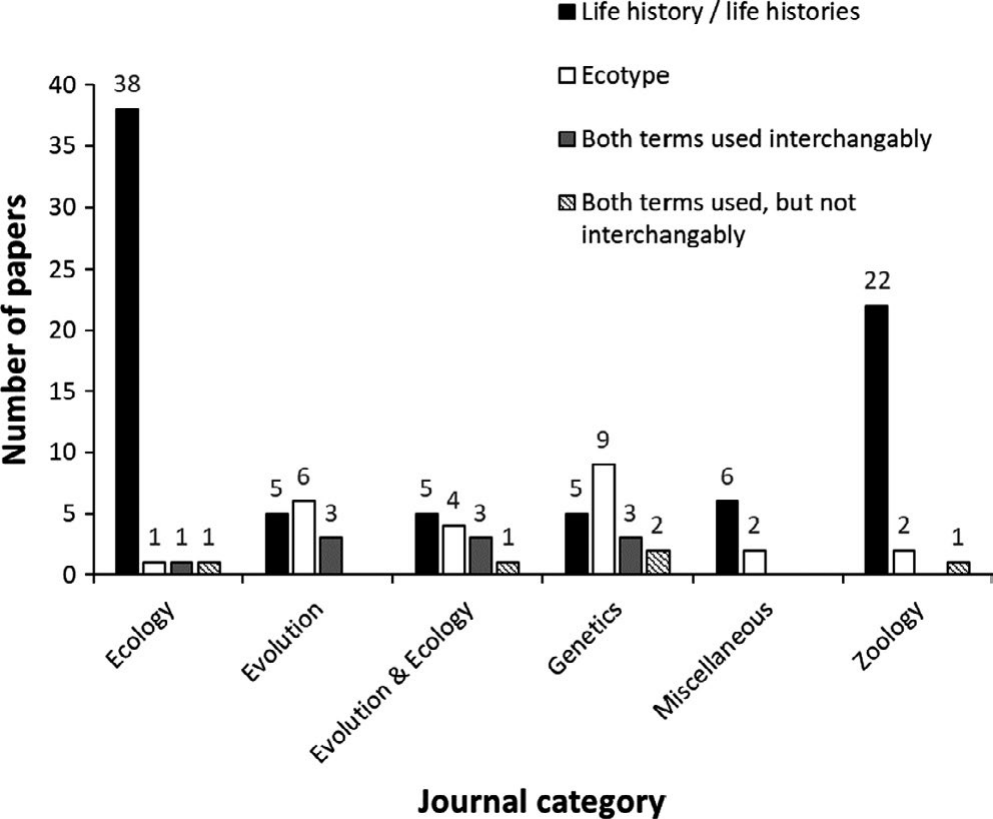
Number of papers that used terms to describe intraspecific diversity in fishes. These data are the combined results of literature searches for sticklebacks, *Oncorhynchus* spp., and lampreys for 1990–2019. The numbers above the bars indicate the number of papers by journal category (as per Table 3). These data indicate that organismal‐ and ecology‐focused journals tended to use the term(s) “life history/life histories.” By contrast, evolution‐ and genetic‐focused journals tended to use the term “ecotype(s).”

**FIGURE 2:**
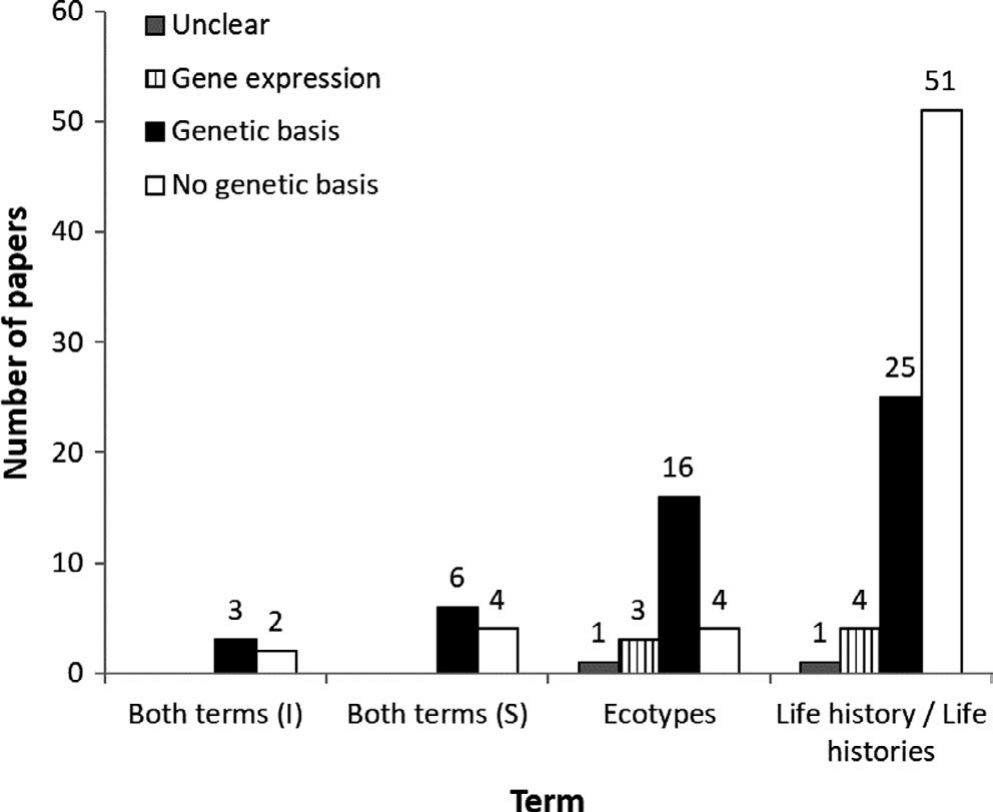
Number of papers that used terms to describe intraspecific diversity in fishes. This data are the combined results of literature searches for sticklebacks, *Oncorhynchus* spp., and lampreys for 1990–2019. “Both terms (I)” = both terms were used independently. “Both terms (S)” = both terms were used synonymously. Numbers above the bars indicate the number of paper by term. These data indicate that studies that used the term “ecotype(s)” tended to find a genetic basis in the diversity examined. By contrast, papers that used the term(s) “life history/life histories” did not tend to report a genetic basis for the diversity examined

**FIGURE 3:**
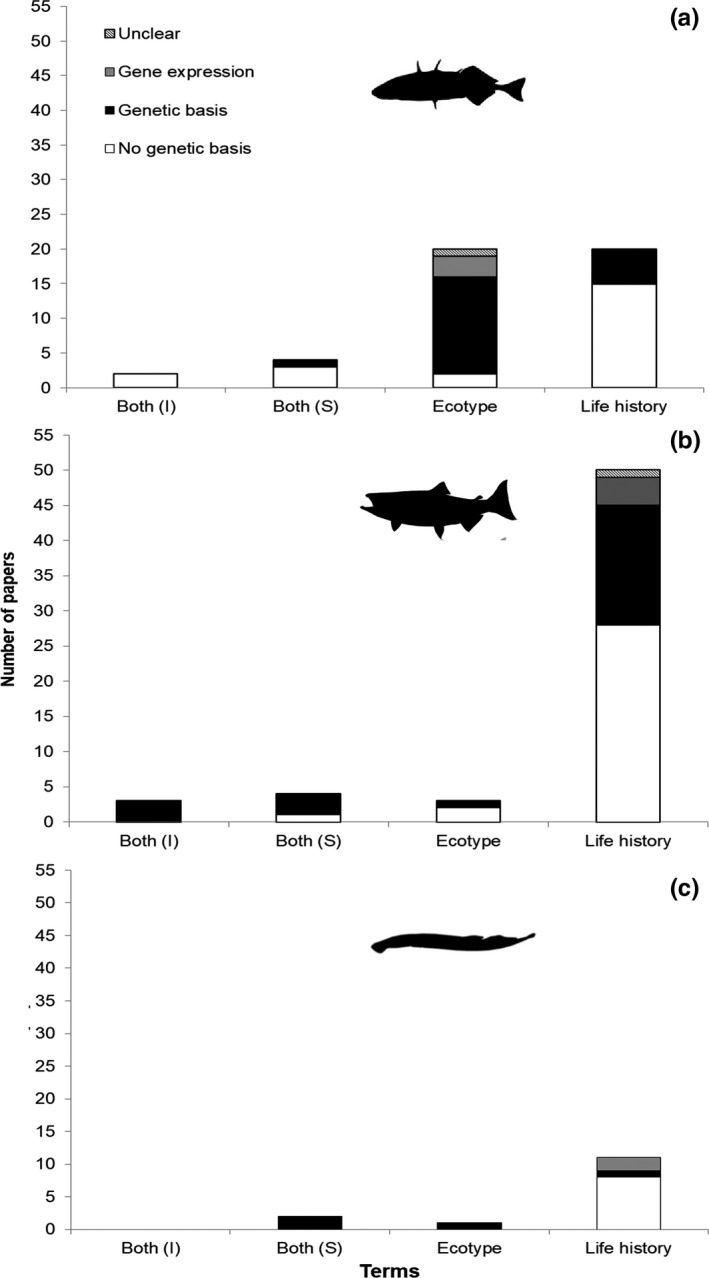
Number of papers that used terms to describe intraspecific diversity in sticklebacks, *Oncorhynchus* spp., and lampreys for 1990–2019. Trends in term use, by taxa. “Both terms (I)” = both terms were used independently. “Both terms (S)” = both terms were used synonymously. These data indicate that the terms “ecotype(s)” and “life history/life histories” were used equally among studies on sticklebacks (a). By contrast, the term “life history/life histories” was used most among studies on *Oncorhynchus* spp. (b) and lampreys (c)

Our literature search yielded 120 articles, including 46 that focused on sticklebacks, 61 on PST, and 13 on lampreys (Table 2). These 120 articles were from 46 different journals that can be categorized into each of six disciplines, including “Ecology,” “Evolution,” “Ecology and Evolution,” “Genetics,” “Miscellaneous,” and “Zoology” (Table 3). Nine articles found by the Web of Science literature search were omitted from our analyses because these papers focused on life stage differences rather than intraspecific differences. Journals with a general focus on organismal biology and ecology tended to use the term(s) “life history/life histories,” whereas journals focusing on evolution and genetics tended to use the term “ecotype(s)” (Figure 1). Studies that used the term ecotype(s) tended to report a genetic basis for the phenotypic differences (Figure 2). The literature on sticklebacks tended to use both life history/life histories and ecotype(s) in equal amounts (Figure 3a), whereas the literature on PST and lampreys tended to use life history/life histories to a greater extent (Figure 3b,c). Taken together, this information suggests that sticklebacks have been a field and laboratory model for evolutionary and genetic research, whereas PST have tended to be the focus of fisheries‐related research and management, and lampreys have experienced comparatively much less research.

**TABLE 3:** Categorization of the journals from which the literature in Table 2 was reviewed

Journal	Category
Acta Oecologia	Ecology
California Fish and Game	Ecology
Canadian Journal of Fisheries and Aquatic Sciences	Ecology
Canadian Journal of Fisheries and Aquatic Sciences Sci Tech	Ecology
Fisheries Research	Ecology
Fisheries Science	Ecology
Journal of Animal Ecology	Ecology
Journal of Fisheries and Wildlife Management	Ecology
Journal of Freshwater Ecology	Ecology
Oecologia	Ecology
Oikos	Ecology
The American Midland Naturalist	Ecology
Transactions of the American Fisheries Society	Ecology
Biological Journal of the Linnean Society	Evolution
Evolutionary Applications	Evolution
Evolution	Evolution
Evolutionary Applications	Evolution
Journal of Evolutionary Biology	Evolution
Biology Letters	Evolution and Ecology
Evolutionary Ecology	Evolution and Ecology
Evolutionary Ecology Research	Evolution and Ecology
Proceedings of the Royal Society B: Biological Sciences	Evolution and Ecology
The American Naturalist	Evolution and Ecology
Acta Naturae	Genetics
Conservation Genetics	Genetics
Genes, Genomes, Genetics	Genetics
Genetica	Genetics
Genome Biology and Evolution	Genetics
Heredity	Genetics
Journal of Heredity	Genetics
Molecular Ecology	Genetics
Molecular Ecology Resources	Genetics
Annales de Limnologie—International Journal of Limnology	Miscellaneous
Earth and Planetary Science Letters	Miscellaneous
Environmental Toxicology and Chemistry	Miscellaneous
Marine Biology	Miscellaneous
Parasites and Vectors	Miscellaneous
PLOS One	Miscellaneous
Behavior	Zoology
Canadian Journal of Zoology	Zoology
Copeia	Zoology
Ecology of Freshwater Fish	Zoology
Environmental Biology of Fishes	Zoology
General and Comparative Endocrinology	Zoology
Journal of Fish Biology	Zoology
Turkish Journal of Zoology	Zoology

The use of terms in our systematic search affected the results that we report. Our search tended to capture ISD, as evidenced by the 69.2% of the 120 papers that focused on this aspect. The other 30.8% included aspects of life history. The literature search for sticklebacks retrieved the highest percentage of papers dealing with ISD (78.3% of the 46 papers), followed by lampreys (71.4% of the 14 papers), and then PST (61.7% of the 60 papers). The reviewers of this paper identified some key papers that were missed with our search (i.e., PST: Bourret et al., 2016; lampreys: Docker et al., 2012; Neave et al., 2019; Rougemont et al., 2015). It is not clear how Bourret et al. (2016) would have been missed because “life history” is in the title of that paper. However, in the other papers “ecotypes” was included as a keyword (Docker et al., 2012; Neave et al., 2019) or in the running title (Rougemont et al., 2015), rather than in the title. In other instances, use of the word “ecotypic” rather than “ecotype” (e.g., Keeley et al., 2005, 2007) resulted in missing papers on PST. It seems likely that other key papers may also have been missed.

### 3.4 Objective 4: Compare definitions with term use and make recommendations

Ecotype originally was used to describe patterns in traits (gene) combinations in particular environments and hence *genotypic* adaptations to particular environments. This term is often used in genetic‐ and evolution‐based journals during 1990–2019. Thus, a general consistency between the classical definition and the current use of ecotype exists. Ecotype would therefore be an obvious term for geneticists and evolutionary biologists wishing to address patterns in genes associated with particular habitats. By contrast, life history and life‐history pathway describe the *phenotypic* responses of organisms to environments and include biological parameters that affect population growth or decline. Thus, the general trend for use of life history in organismal‐ and ecology‐based journals during 1990–2019 makes sense. Life‐history types imply discontinuity in phenotypic expressions, whereas life‐history pathways (e.g., Thorpe et al., 1998) imply continuity in phenotypic expression (Table 1).

Although we did not assess the use of derivative terms such as morphotypes, ecomorphotypes, ecophenotypes, these terms arguably do not offer insight into ISD, and we therefore recommend that authors either should not use these terms or use them sparingly. All attempts to describe ISD would benefit from clear definitions. Ecotypes must show genotypic adaptations—but not enough to warrant calling the genotypes distinct species. Life histories/life‐history pathways, by contrast, describe phenotypic responses in demographic parameters (e.g., Winemiller, 2005). Therefore, it is reasonable to use the terms life history or life‐history pathway to describe recognizable life‐history traits. In situations where the genetic or life‐history components of ISD are not well understood, a conservative approach would be to simply call them phenotypes.

## 4 CONCLUSIONS

Understanding and preserving ISD is important for species conservation. Ecotype was originally used to describe genotypic adaptation to environments, and recent studies generally use this term in a similar way. By contrast, life history includes biological parameters that affect abundance and population growth and decline, and recent studies generally use this term in a similar way. Ecotype and life history were used equally among recent studies on sticklebacks. By contrast, life history was used more frequently than ecotype among recent studies on PST and lampreys.

## CONFLICT OF INTEREST

None declared.

## AUTHOR CONTRIBUTIONS

**Benjamin J. Clemens:** Conceptualization (lead); Data curation (lead); Formal analysis (lead); Investigation (lead); Methodology (lead); Resources (lead); Validation (lead); Visualization (lead); Writing‐original draft (lead); Writing‐review & editing (lead). **Carl Schreck:** Conceptualization (supporting); Resources (equal); Supervision (supporting); Writing‐original draft (supporting); Writing‐review & editing (supporting).

## Data Availability

No data were archived for this paper. All data are included within the manuscript.

## References

[ece37884-bib-0001] Arai, T., Chino, N., & Goto, A. (2010). Life history variation among years in a brackish water type of the nine spine stickleback, *Pungitius pungitius* . Environmental Biology of Fishes, 88, 319–322.

[ece37884-bib-0002] Arai, T., & Goto, A. (2005). Flexible life history strategies of ninespine sticklebacks, genus *Pungitius* . Environmental Biology of Fishes, 74, 43–50. 10.1007/s10641-005-3221-5

[ece37884-bib-0003] Arismendi, I., Sanzana, J., & Soto, D. (2011). Seasonal age distributions and maturity stage in a naturalized rainbow trout (*Oncorhynchus mykiss* Walbaum) population in southern Chile reveal an ad‐fluvial life history. Annales de Limnologie ‐ International Journal of Limnology, 47, 133–140.

[ece37884-bib-0004] Arostegui, M. C., Quinn, T. P., Seeb, L. W., Seeb, J. E., & McKinney, G. J. (2018). Retention of a chromosomal inversion from an anadromous ancestor provides the genetic basis for alternative freshwater ecotypes in rainbow trout. Molecular Ecology, 28, 1412–1427. 10.1111/mec.15037 30714254

[ece37884-bib-0005] Endangered Species Act . (1973). (16 U.S.C. 1531‐1544, 87 Stat. 884), as amended ‐‐ Public Law 93‐205, approved December 28, 1973, repealed the Endangered Species Conservation Act of December 5, 1969 (P.L. 91‐135, 83 Stat. 275). The 1969 Act had amended the Endangered Species Preservation Act of October 15, 1966 (P.L. 89‐669, 80 Stat. 926).

[ece37884-bib-0006] Baker, J. A., Cresko, W. A., Foster, S. A., & Heins, D. C. (2005). Life‐history differentiation of benthic and limnetic ecotypes in a polytypic population of threespine stickleback (*Gasterosteus aculeatus*). Evolutionary Ecology Research, 7, 121–131.

[ece37884-bib-0007] Baker, J. A., & Foster, S. A. (2002). Phenotypic plasticity for life history traits in a stream population of the threespine stickleback, *Gasterosteus aculeatus* L. Ecology of Freshwater Fish, 11, 20–29.

[ece37884-bib-0008] Baker, J. A., Foster, S. A., Heins, D. C., Bell, M. A., & King, R. W. (1998). Variation in female life‐history traits among Alaskan populations of the threespine stickleback, *Gasterosteus aculeatus* L. (Pisces: Gasterosteidae). Biological Journal of the Linnean Society, 63, 141–159. 9480735

[ece37884-bib-0009] Baker, J. A., Heins, D. C., & Baum, J. E. (2019). Trajectory and rate of change in female life‐history traits following colonization of a freshwater, lacustrine environment by oceanic threespine stickleback. Evolutionary Ecology Research, 20, 247–263.

[ece37884-bib-0010] Baker, J. A., Heins, D. C., Foster, S. A., & King, R. W. (2008). An overview of life‐history variation in female threespine stickleback. Behaviour, 145, 579–602.

[ece37884-bib-0011] Baker, J. A., Heins, D. C., King, R. W., & Foster, S. A. (2011). Rapid shifts in multiple life history traits in a population of threespine stickleback. Journal of Evolutionary Biology, 24, 863–870. 21276108 10.1111/j.1420-9101.2010.02217.x

[ece37884-bib-0012] Baker, J. A., Räsänen, K., Moore, J.‐S., & Hendry, A. P. (2013). Genetic and plastic contributions to trait divergence between parapatric habitats: Female life history traits in threespine stickleback within the Misty Lake system. Evolutionary Ecology Research, 15, 473–487.

[ece37884-bib-0013] Beaulaton, L., Taverny, C., & Castelnaud, G. (2008). Fishing, abundance, and life history traits of the anadromous sea lamprey (*Petromyzon marinus*) in Europe. Fisheries Research, 92, 90–101.

[ece37884-bib-0014] Beckman, B. R., Gadberry, B., Parkins, P., Cooper, K. A., & Arkush, K. D. (2007). State‐dependent life history plasticity in Sacramento River winter‐run Chinook salmon (*Oncorhynchus tshawytscha*): Interactions among photoperiod and growth modulate smolting and early male maturation. Canadian Journal of Fisheries and Aquatic Sciences, 64, 256–271.

[ece37884-bib-0015] Behnke, R. J. (2002). Trout and salmon of North America. The Free Press.

[ece37884-bib-0016] Bell, M. A., & Foster, S. A. (1994). The evolutionary biology of the threespine stickleback. Oxford University Press.

[ece37884-bib-0017] Benjamin, J. R., Connolly, P. J., Romine, J. G., & Perry, R. W. (2013). Potential effects of changes in temperature and food resources on life history trajectories of juvenile *Oncorhynchus mykiss* . Transactions of the American Fisheries Society, 142, 208–220.

[ece37884-bib-0018] Berejikian, B. A., Bush, R. A., & Campbell, L. A. (2014). Maternal control over offspring life history in a partially anadromous species, *Oncorhynchus mykiss* . Transactions of the American Fisheries Society, 143, 369–379.

[ece37884-bib-0019] Bergstedt, R. A., & Seelye, J. G. (1995). Evidence for lack of homing by sea lampreys. Transactions of the American Fisheries Society, 124, 235–239.

[ece37884-bib-0020] Berner, D., Kaeuffer, R., Grandchamp, A.‐C., Raeymaekers, J. A. M., Räsänen, K., & Hendry, A. P. (2011). Quantitative genetic inheritance of morphological divergence in a lake‐stream stickleback ecotype pair: Implications for reproductive isolation. Journal of Evolutionary Biology, 24, 1975–1983. 10.1111/j.1420-9101.2011.02330.x 21649765

[ece37884-bib-0021] Boguski, D. A., Reid, S. B., Goodman, D. H., & Docker, M. F. (2012). Genetic diversity, endemism and phylogeny of lampreys within the genus *Lampetra sensu stricto* (Petromyzontiformes: Petromyzontidae) in western North America. Journal of Fish Biology, 81, 1891–1914. 23130690 10.1111/j.1095-8649.2012.03417.x

[ece37884-bib-0022] Bourret, S. L., Caudill, C. C., & Keefer, M. L. (2016). Diversity of juvenile Chinook salmon life history pathways. Reviews in Fish Biology and Fisheries, 26, 375–403. 10.1007/s11160-016-9432-3

[ece37884-bib-0023] Brannon, E. L., Powell, M. S., Quinn, T. P., & Talbot, A. (2004). Population structure of Columbia River Basin Chinook salmon and steelhead trout. Reviews in Fisheries Science, 12, 99–232. 10.1080/10641260490280313

[ece37884-bib-0024] Bryan, M. B., Zalinski, D., Filcek, K. B., Libants, S., Li, W., & Scribner, K. T. (2005). Patterns of invasion and colonization of the sea lamprey (*Petromyzon marinus*) in North America as revealed by microsatellite genotypes. Molecular Ecology, 14, 3757–3773. 10.1111/j.1365-294X.2005.02716.x 16202094

[ece37884-bib-0025] Carlson, S. M., Rich, H. B. Jr, & Quinn, T. P. (2004). Reproductive life‐span and sources of mortality for alternative life history strategies in sockeye salmon, *Oncorhynchus nerka* . Canadian Journal of Zoology, 82, 1878–1885.

[ece37884-bib-0026] Carlson, S. M., & Seamons, T. R. (2008). A review of quantitative genetic components of fitness in salmonids: Implications for adaptation to future change. Evolutionary Applications, 1, 222–238. 10.1111/j.1752-4571.2008.00025.x 25567628 PMC3352437

[ece37884-bib-0027] Chavarie, L., Howland, K. L., & Tonn, W. M. (2013). Sympatric polymorphism in Lake Trout: The coexistence of multiple shallow‐water morphotypes in Great Bear Lake. Transactions of the American Fisheries Society, 142, 814–823. 10.1080/00028487.2013.763855

[ece37884-bib-0028] Clarke, W. C., Withler, R. E., & Shelbourn, J. E. (1992). Genetic control of juvenile life history patterns in Chinook salmon (*Oncorhynchus tshawytscha*). Canadian Journal of Fisheries and Aquatic Sciences, 49, 2300–2306.

[ece37884-bib-0029] Clemens, B. J., van de Wetering, S., Sower, S. A., & Schreck, C. B. (2013). Maturation characteristics and life‐history strategies of the Pacific lamprey, *Entosphenus tridentatus* . Canadian Journal of Zoology, 91, 775–788.

[ece37884-bib-0030] Collyer, M. L., Hall, M. E., Smith, M. D., & Hoagstrom, C. W. (2015). Habitat‐morphotype associations of Pecos pupfish (*Cyprinodon pecosensis*) in isolated habitat complexes. Copeia, 2015, 181–199.

[ece37884-bib-0031] Copeland, T., & Venditti, D. A. (2009). Contribution of three life history types to smolt production in a Chinook salmon (*Oncorhynchus tshawytscha*) population. Canadian Journal of Fisheries and Aquatic Sciences, 66, 1658–1665. 10.1139/F09-110

[ece37884-bib-0032] COSEWIC (Committee on the Status of Endangered Wildlife in Canada). (2021). COSEWIC guidelines for recognizing designatable units. Retrieved from https://cosewic.ca/index.php/en‐ca/reports/preparing‐status‐reports/guidelines‐recognizing‐designatable‐units

[ece37884-bib-0033] Craig, B. E., Simenstad, C. A., & Bottom, D. L. (2014). Rearing in natural and recovering tidal wetlands enhances growth and life history diversity of Columbia estuary tributary coho salmon *ONcorhynchus kisutch* population. Journal of Fish Biology, 85, 31–51. 24890886 10.1111/jfb.12433

[ece37884-bib-0034] Cruz‐Font, L., Shuter, B. J., Blanchfield, P. J., Minns, C. K., & Rennie, M. D. (2019). Life at the top: Lake ecotype influences the foraging pattern, metabolic costs and life history of an apex fish predator. Journal of Animal Ecology, 88, 702–716. 10.1111/1365-2656.12956 30712263

[ece37884-bib-0035] de Queiroz, K. (2020). An updated concept of subspecies resolves a dispute about the taxonomy of incompletely separated lineages. Herpetological Review, 51, 459–461.

[ece37884-bib-0036] DeWitt, T. J., & Scheiner, S. M. (2004). Phenotypic variation from single phenotypes: A primer. In: T. J. DeWitt, & S. M. Scheiner (Eds.), Phenotypic plasticity: Functional and conceptual approaches (pp. 1–9). Oxford University Press Inc.

[ece37884-bib-0037] Docker, M. F. (2009). A review of the evolution of non‐parasitism in lampreys and an update of the paried species concept. American Fisheries Society Symposium, 72, 71–114.

[ece37884-bib-0038] Docker, M. F. (2015). Lampreys: biology, conservation and control. Fish and Fisheries Series, Vol. 37. Springer, Dordrecht. The Netherlands.

[ece37884-bib-0039] Docker, M. F. (2019). Lampreys: Biology, conservation and control. Fish and Fisheries Series, Vol. 38. Springer, Dordrecht, The Netherlands.

[ece37884-bib-0040] Docker, M. F., Hume, J., & Clemens, B. J. (2015). Introduction: A surfeit of lampreys. In M. F. Docker (Ed.), Lampreys: Biology, conservation and control, (Vol. 1, pp. 1–34). Fish and Fisheries Monograph Series, Springer.

[ece37884-bib-0041] Docker, M. F., Mandrak, N. E., & Heath, D. D. (2012). Contemporary gene flow between “paired” silver (*Ichthyomyzon unicuspis*) and northern brook (*I. fossor*) lampreys: Implications for conservation. Conservation Genetics, 13, 823–835. 10.1007/s10592-012-0332-3

[ece37884-bib-0042] Docker, M. F., & Potter, I. C. (2019). Life history evolution in lampreys: Alternative migratory and feedings types. In M. F. Docker (Ed.), Lampreys: Biology, conservation and control. (Vol. 2, pp. 287–410). Fish and Fisheries Series, Vol. 38, Springer.

[ece37884-bib-0043] Eizaguirre, C., Lenz, T. L., Sommerfeld, R. D., Harrod, C., Kalbe, M., & Milinski, M. (2011). Parasite diversity, patterns of MHC II variation and olfactory based mate choice in diverging three‐spined stickleback ecotypes. Evolutionary Ecology, 25, 605–622.

[ece37884-bib-0044] Esteve, M., & McLennan, D. A. (2007). The phylogeny of *Oncorhynchus* (Euteleostei: Salmonidae) based on behavioral and life history characters. Copeia, 2007, 520–533.

[ece37884-bib-0045] Evans, M. L., Neff, B. D., & Heath, D. D. (2010). Quantitative genetic and translocation experiments reveal genotype‐by‐environment effects on juvenile life‐history traits in two populations of Chinook salmon (*Oncorhynchus tshawytscha*). Journal of Evolutionary Biology, 23, 687–698. 20102438 10.1111/j.1420-9101.2010.01934.x

[ece37884-bib-0046] Evans, T. M., & Bauer, J. E. (2016). Using stable isotopes and C : N ratios to examine the life‐history strategies and nutritional sources of larval lampreys. Journal of Fish Biology, 88, 638–654. 26707340 10.1111/jfb.12858

[ece37884-bib-0047] Evans, T. M., & Limburg, K. E. (2019). Parasitism offers large rewards but carries high risks: Predicting parasitic strategies under different life history conditions in lampreys. Journal of Evolutionary Biology, 32, 794–805. 10.1111/jeb.13481 31021026

[ece37884-bib-0048] Faukner, J., Silloway, S., Sparkman, M., & Drobny, P. (2017). A previously undocumented life history behavior in juvenile coho salmon (*Oncorhynchus kisutch*) from the Klamath River, California. California Fish and Game, 103, 72–78.

[ece37884-bib-0049] Fillatre, E. K., Etherton, P., & Heath, D. D. (2003). Bimodal run distribution in a northern population of sockeye salmon (*Oncorhynchus nerka*): Life history and genetic analysis on a temporal scale. Molecular Ecology, 12, 1793–1805. 12803632 10.1046/j.1365-294x.2003.01869.x

[ece37884-bib-0050] Fleming, I. A., & Reynolds, J. D. (2004). Salmonid breeding systems. In A. D. Hendry, & S. C. Stearns (Eds.), Evolution illuminated: Salmon and their relatives (pp. 264–294). Oxford University Press.

[ece37884-bib-0051] Ford, M. J. (2004). Conservation units and preserving diversity. In A. D. Hendry, & S. C. Stearns (Eds.), Evolution illuminated: Salmon and their relatives (pp. 338–357). Oxford University Press.

[ece37884-bib-0052] Forest, A. R., Semeniuk, C. A. D., Heath, D. D., & Pitcher, T. E. (2016). Additive and non‐additive genetic components of the jack male life history in Chinook salmon (*Oncorhynchus tshawytscha*). Genetica, 144, 477–485. 10.1007/s10709-016-9917-y 27450674

[ece37884-bib-0053] Foster, S. A., Baker, J. A., & Bell, M. A. (1992). Phenotypic integration of life history and morphology: An example from the three‐spined stickleback, *Gasterosteus aculeatus* L. Journal of Fish Biology, 41(Suppl. B), 21–35.

[ece37884-bib-0054] Gahr, C. L., Boehm, T., & Milinski, M. (2018). Female assortative mate choice functionally validates synthesized male odours of evolving stickleback river‐lake ecotypes. Biology Letters, 14, 20180730. 10.1098/rsbl.2018.0730 30958253 PMC6303515

[ece37884-bib-0055] Gess, R., Coates, M. I., & Rubidge, B. S. (2006). A lamprey from the Devonian period of South Africa. Nature, 443, 981–984. 10.1038/nature05150 17066033

[ece37884-bib-0056] Gibbons, T. C., Metzger, D. C. H., Healy, T. M., & Schulte, P. M. (2017). Gene expression plasticity in response to salinity acclimation in threespine stickleback ecotypes from different salinity habitats. Molecular Ecology, 26, 2711–2725. 10.1111/mec.14065 28214359

[ece37884-bib-0057] Gibbons, T. C., Rudman, S. M., & Schulte, P. M. (2016). Responses to simulated winter conditions differ between threespine stickleback ecotypes. Molecular Ecology, 25, 764–775. 10.1111/mec.13507 26645643

[ece37884-bib-0058] Ginsburg, I. (1937). The species and its subdivisions. Copeia, 1937, 184–188. 10.2307/1436140

[ece37884-bib-0059] Godbout, L., Trudel, M., Irvine, J. R., Wood, C. C., Grove, M. J., Schmitt, A. K., & McKeegan, K. D. (2010). Sulfur isotopes in otoliths allow discrimination of anadromous and non‐anadromous ecotypes of sockeye salmon (*Oncorhynchus nerka*). Environmental Biology of Fishes, 89, 521–532. 10.1007/s10641-010-9689-7

[ece37884-bib-0060] Gregor, J. W. (1944). The ecotype. Biological Reviews, 19, 20–30. 10.1111/j.1469-185X.1944.tb00299.x

[ece37884-bib-0061] Gresswell, R. E., Liss, W. J., & Larson, G. L. (1994). Life‐history organization of Yellowstone cutthroat trout (*Oncorhynchus clarki bouvieri*) in Yellowstone Lake. Canadian Journal of Fisheries and Aquatic Sciences, 51(Suppl. 1), 298–309.

[ece37884-bib-0062] Groot, C., & Margolis, L. (1991). Pacific salmon life histories. UBC Press.

[ece37884-bib-0063] Haig, S. M., Beever, E. A., Chambers, S. M., Draheim, H. M., Dugger, B. D., Dunham, S. M., Elliott‐Smith, E., Fontaine, J. B., Kesler, D. C., Knaus, B. J., Lopes, I. F., Loschl, P., Mullins, T. D., & Sheffield, L. M. (2006). Taxonomic considerations in listing subspecies under the U. S. Endangered Species Act. Conservation Biology, 20, 1584–1594. 17181793 10.1111/j.1523-1739.2006.00530.x

[ece37884-bib-0064] Hale, M. C., McKinney, G. J., Thrower, F. P., & Nichols, K. M. (2018). Evidence of sex‐bias in gene expression in the brain transriptome of two populations of rainbow trout (*Oncorhynchus mykiss*) with divergent life histories. Public Library of Science One, 13, e0193009. 29447294 10.1371/journal.pone.0193009 PMC5814004

[ece37884-bib-0065] Hale, M. C., Thrower, F. P., Berntson, E. A., Miller, M. R., & Nichols, K. M. (2013). Evaluating adaptive divergence between migratory and nonmigratory ecotypes of a salmonid fish, *Oncorhynchus mykiss* . Genes, Genomes, Genetics, 3, 1273–1285. 23797103 10.1534/g3.113.006817 PMC3737167

[ece37884-bib-0066] Haley, A. L., Dalziel, A. C., & Weir, L. K. (2019). A comparison of nuptial coloration and breeding behaviour in white and common marine threespine stickleback (*Gasterosteus aculeatus*) ecotypes. Evolutioanry Ecology Research, 20, 145–166.

[ece37884-bib-0067] Hanson, D., Moore, J.‐S., Taylor, E. B., Barrett, R. D. H., & Hendry, A. P. (2016). Assessing reproductive isolation using a contact zone between parapatric lake‐stream stickleback ecotypes. Journal of Evolutionary Biology, 29, 2491–2501. 10.1111/jeb.12978 27633750

[ece37884-bib-0068] Hanson, K. C., Gale, W. L., Simpson, W. G., Kennedy, B. M., & Ostrand, K. G. (2011). Physiological characterization of hatchery‐origin juveile steelhead *Oncorhynchus mykiss* adopting divergent life history‐strategies. Journal of Fish and Wildlife Management, 2, 61–71.

[ece37884-bib-0069] Hardisty, M. W. (2006). Lampreys: Life without jaws. Forrest Text.

[ece37884-bib-0070] Healey, M. C. (1991). Life history of Chinook salmon (*Oncorhynchus tshawytscha*). In C. Groot, & L. Margolis (Eds.), Pacific salmon life histories (pp. 311–393). University of British Columbia Press.

[ece37884-bib-0071] Heath, D. D., Rankin, L., Bryden, C. A., Heath, J. W., & Shrimpton, J. M. (2002). Heritability and Y‐chromosome influence in the jack male life history of Chinook salmon (*Oncorhynchus tshawytscha*). Heredity, 89, 311–317. 10.1038/sj.hdy.6800141 12242648

[ece37884-bib-0072] Heins, D. C. (2019). Life history variation within and among populations of the ninespine stickleback (*Pungitius pungitius*) in Alaska: Lake‐stream constrasts. The American Midland Naturalist, 182, 228–238.

[ece37884-bib-0073] Heins, D. C., Knoper, J., & Baker, J. A. (2016). Consumptive and non‐consumptive effects of predation by introducted northern pike on life history traits in threespine stickleback. Evolutionary Ecology Research, 17, 355–372.

[ece37884-bib-0074] Hendry, A. P. (2009). Ecological speciation! Or the lack thereof? Canadian Journal of Fisheries and Aquatic Sciences, 66, 1383–1398.

[ece37884-bib-0075] Hendry, A. P., Bohlin, T., Jonsson, B., & Berg, O. K. (2004b). To sea or not to sea? Anadromy versus non‐anadromy in salmonids. In A. D. Hendry, & S. C. Stearns (Eds.), Evolution illuminated: Salmon and their relatives (pp. 92–125). Oxford University Press.

[ece37884-bib-0076] Hendry, A. P., Bolnick, D. I., Berner, D., & Peichel, C. L. (2009). Along the speciation continuum in sticklebacks. Journal of Fish Biology, 75, 2000–2036. 10.1111/j.1095-8649.2009.02419.x 20738669

[ece37884-bib-0077] Hendry, A. P., Castric, V., Kinnison, M. T., & Quinn, T. P. (2004a). The evolution of philopatry and dispersal Homing versus straying in salmonids. In A. D. Hendry, & S. C. Stearns (Eds.), Evolution illuminated: Salmon and their relatives (pp. 52–91). Oxford University Press.

[ece37884-bib-0078] Hendry, A. P., Peichel, C. L., Matthews, B., Boughman, J. W., & Nosil, P. (2013). Stickleback research: The now and the next. Evolutionary Ecology Research, 15, 111–141.

[ece37884-bib-0079] Hendry, A. P., & Quinn, T. P. (1997). Variation in adult life historyz and morphology among Lake Washington sockeye salmon (*Oncorhynchus nerka*) populations in relation to habitat features and ancestral affinities. Canadian Journal of Fisheries and Aquatic Sciences, 54, 75–84.

[ece37884-bib-0080] Hendry, A. P., & Stearns, S. C. (2004). Evolution illuminated: Salmon and their relatives. Oxford University Press.

[ece37884-bib-0081] Huang, Y., Feulner, P. G. D., Eizaguirre, C., Lenz, T. L., Bornberg‐Bauer, E., Milinski, M., Reusch, T. B. H., & Chain, F. J. J. (2019). Genome‐wide genotype expression relationships reveal both copy number and single nucleotide differentiation contribute to differential gene expression between stickleback ecotypes. Genome Biology and Evolution, 11, 2344–2359. 31298693 10.1093/gbe/evz148 PMC6735750

[ece37884-bib-0082] Hubbs, C. L. (1943). Criteria for subspecies, species and genera, as determined by researchers on fishes. Annals of the New York Academy of Sciences, Criteria for Vertebrate Subspecies, Species and Genera, 44, 109–121.

[ece37884-bib-0083] Hufford, K. M., & Mazer, S. J. (2003). Plant ecotypes: Genetic differentiation in the age of ecological restoration. Trends in Ecology and Evolution, 18, 147–155.

[ece37884-bib-0084] Hume, J. B., Adams, C. E., Mable, B., & Bean, C. W. (2013). Sneaker male mating tactics between lampreys (Petromyzontiformes) exhibiting alternative life history strategies. Journal of Fish Biology, 82, 1093–1100. 23464566 10.1111/jfb.12047

[ece37884-bib-0085] Hume, J. B., Recknagel, H., Bean, C. W., Adams, C. E., & Mable, B. K. (2018). RADseq and mate choice assays reveal unidirectional gene flow among three lamprey ecotypes despite weak assortative mating: Insights into the formation and stability of multiple ecotypes in sympatry. Molecular Ecology, 27, 4572–4590. 30252984 10.1111/mec.14881

[ece37884-bib-0086] Hutchings, J. A. (2004). Norms of reactoin and phenotypic plasticity in salmonid life histories. In A. P. Hendry, & S. C. Stearns (Eds.), Evolution illuminated: Salmon and their relatives (pp. 154–174). Oxford University Press.

[ece37884-bib-0087] Ishikawa, A., Kusakabe, M., Yoshida, K., Ravinet, M., Makino, T., Toyoda, A., Fujiyama, A., & Kitano, J. (2017). Different contributions of local‐ and distant‐regulatory changes to transriptome divergence between stickleback ecotypes. Evolution, 71, 565–581. 28075479 10.1111/evo.13175

[ece37884-bib-0088] Jamie, G. A., & Meier, J. I. (2020). The persistence of polymorphisms across species radiations. Trends in Ecology and Evolution, 35, 795–808. 10.1016/j.tree.2020.04.007 32408996

[ece37884-bib-0089] Janvier, P. (2008). Early jawless vertebrates and cyclostome origins. Zoological Science, 25, 1045–1056. 10.2108/zsj.25.1045 19267641

[ece37884-bib-0090] Johnson, S. W., Thedinga, J. F., & Koski, K. V. (1992). Life history of juvenile ocean‐type Chinook salmon (*Oncorhynchus tshawytscha*) in the Situk River, Alaska. Canadian Journal of Fisheries and Aquatic Sciences, 49, 2621–2629.

[ece37884-bib-0091] Jones, K. K., Cornwell, T. J., Bottom, D. L., Campbell, L. A., & Stein, S. (2014). The contribution of estuary‐resident life histories to the return of adult *Oncorhynchus kisutch* . Journal of Fish Biology, 85, 52–80. 24766645 10.1111/jfb.12380

[ece37884-bib-0092] Karve, A. D., Baker, J. A., & von Hippel, F. A. (2013). Female life history traits of a species pair of threespine stickleback in Mud Lake, Alaska. Evolutionary Ecology Research, 15, 171–187.

[ece37884-bib-0093] Kaufman, J., Lenz, T. L., Kalbe, M., Milinski, M., & Eizaguirre, C. (2017). A field reciprocal transplant experiment reveals asymmetric costs of migration between lake and river ecotypes of three‐spined stickleback (*Gastoerosteus aculeatus*). Journal of Evolutionary Biology, 30, 938–950. 28211194 10.1111/jeb.13057

[ece37884-bib-0094] Keeley, E. R., Parkinson, E. A., & Taylor, E. B. (2005). Ecotypic differentiation of native rainbow trout (*Oncorhynchus mykiss*) populations from British Columbia. Canadian Journal of Fisheries and Aquatic Sciences, 62, 1523–1539.

[ece37884-bib-0095] Keeley, E. R., Parkinson, E. A., & Taylor, E. B. (2007). The origins of ecotypic variation of rainbow trout: A test of environmental vs. genetically based differences in morphology. Journal of Evolutionary Biology, 20, 725–736. 10.1111/j.1420-9101.2006.01240.x 17305838

[ece37884-bib-0096] Kim, S.‐Y., Costa, M. M., Esteve‐Codina, A., & Velando, A. (2017). Transriptional mechanisms underlying life‐history responses to climate change in the three‐spined stickleback. Evolutionary Applications, 10, 718–730. 28717391 10.1111/eva.12487 PMC5511362

[ece37884-bib-0097] Kinnison, M. T., Quinn, T. P., & Unwin, M. J. (2011). Correlated contemporary evolution of life history traits in New Zealand Chinook salmon, *Oncorhynchus tshawytscha* . Heredity, 106, 448–459. 21224875 10.1038/hdy.2010.162 PMC3131966

[ece37884-bib-0098] Kitano, J., & Lema, S. C. (2013). Divergence in the thyroid hormone concentrations between juveniles of marine and stream ecotypes of the threespine stickleback. Evolutionary Ecology Research, 15, 143–153.

[ece37884-bib-0099] Kloh, J. S., Figueredo, C. C., & Eterovick, P. C. (2019). How close is microhabitat and diet association in aquatic ecomorphotypes? A test with tadpoles of syntopic species. Hydrobiologia, 828, 271–285. 10.1007/s10750-018-3818-2

[ece37884-bib-0100] Kristjánsson, B., Skúlason, S., & Noakes, D. L. G. (2002). Morphological segregation of Icelandic threespine stickleback (*Gasterosteus aculeatus* L). Biological Journal of the Linnean Soccety, 76, 247–257. 10.1046/j.1095-8312.2002.00063.x

[ece37884-bib-0101] Kurz, M. L., Heins, D. C., Bell, M. A., & von Hippel, F. A. (2016). Shifts in life history traits of two introduced populations of threespine sticklebck. Evolutionary Ecology Research, 17, 225–242.

[ece37884-bib-0102] Kusakabe, M., Ishikawa, A., Ravinet, M., Yoshida, K., Makino, T., Toyoda, A., Fujiyama, A., & Kitano, J. (2017). Genetic basis for variation in salinity tolerance between stickleback ecotypes. Molecular Ecology, 26, 304–319. 27706866 10.1111/mec.13875

[ece37884-bib-0103] Lamperth, J. S., Quinn, T. P., & Zimmerman, M. S. (2018). Levels of stored energy but not marine foraging patterns differentiate seasonal ecotypes of wild and hatchery steelhead (*Oncorhynchus mykiss*) returning to the Kalama River, Washinton. Canadian Journal of Fisheries and Aquatic Sciences, 74, 157–167.

[ece37884-bib-0104] Lenz, T., Eizaguirre, C., Rotter, B., Kalbe, M., & Milinski, M. (2013). Exploring local immunological adaptation of two stickleback ecotypes by experimental infection and transcriptome‐wide digital gene expression analysis. Molecular Ecology, 22, 774–786. 10.1111/j.1365-294X.2012.05756.x 22971109 PMC3579235

[ece37884-bib-0105] Lessard, R. B., Hilborn, R., & Chasco, B. E. (2008). Ecapement goal analysis and stock reconstruction of sockeye salmon populations (*Oncorhynchus nerka*) using life‐history models. Canadian Journal of Fisheries and Aquatic Sciences, 65, 2269–2278.

[ece37884-bib-0106] Lessios, H. A., & Weinberg, J. R. (1994). Genetic and morphological divergence among morphotypes of the isopod *Excirolana* on the two sides of the isthmus of Panama. Evolution, 48, 530–548. 28568259 10.1111/j.1558-5646.1994.tb01342.x

[ece37884-bib-0107] Lichatowich, J. (1999). Salmon without rivers: A history of the Pacific salmon crisis. Island Press.

[ece37884-bib-0108] Liu, S., Hansen, M. M., & Jacobsen, M. W. (2016). Region‐wide and ecotype‐specific differences in demographic histories of threespine stickleback populations, estimated from whole genome sequences. Molecular Ecology, 25, 5187–5202. 27569902 10.1111/mec.13827

[ece37884-bib-0109] Lucek, K., Sivasundar, A., Kristjánsson, B. K., Skúlason, S., & Seehausen, O. (2014). Quick divergence but slow convergence during ecotype formation in lake and stream stickleback pairs of variable age. Journal of Evolutionary Biology, 27, 1878–1892. 10.1111/jeb.12439 24976108

[ece37884-bib-0110] Maitland, P. S., Renaud, C. B., Quintella, B. R., Close, D. A., & Docker, M. F. (2015). Conservation of native lampreys. In M. F. Docker (Ed.), Lampreys: Biology, conservation and control (Vol. 1, pp. 375–428). Fish and Fisheries Series, Vol. 37. Springer.

[ece37884-bib-0111] Mayr, E. (1947). Ecological factors in speciation. Evolution, 1, 263–288. 10.1111/j.1558-5646.1947.tb02723.x

[ece37884-bib-0112] McKinnon, J. S., & Rundle, H. D. (2002). Speciation in nature: Thre threespine stickleback model systems. Trends in Ecology and Evolution, 480–488.

[ece37884-bib-0113] Miller, J. A., Butler, V. L., Simenstad, C. A., Backus, D. H., & Kent, A. J. R. (2011). Life history variation in upper Columbia River Chinook salmon (*Oncorhynchus tshawytscha*): A comparison using modern and ~500‐year‐old archaeological otoliths. Canadian Journal of Fisheries and Aquatic Sciences, 68, 603–617.

[ece37884-bib-0114] Miller, S. E., Roesti, M., & Schluter, D. (2019). A single interacting species leads to widespread parallel evolution of the stickleback genome. Current Biology, 29, 530–537. 10.1016/j.cub.2018.12.044 30686736 PMC6371808

[ece37884-bib-0115] Millet, A., Kristjánsson, B. K., Einarsson, Á., & Räsänen, K. (2013). Spatial phenotypic and genetic structure of threespine stickleback (*Gasterosteus aculeatus*) in a heterogeneous natural system, Lake Myvatn, Iceland. Ecology and Evolution, 3, 3219–3232. 24223263 10.1002/ece3.712 PMC3797472

[ece37884-bib-0116] Montgomery, D. R. (2000). Coevolution of the Pacific salmon and Pacific rim topography. Geology, 28, 1107–1110. 10.1130/0091-7613(2000)028<1107:COTPSA>2.3.CO;2

[ece37884-bib-0117] Moreira, A. I., & Taylor, E. B. (2015). The origin and genetic divergence of “black” kokanee, a novel reproductive ecotype of *Oncorhynchus nerka* . Canadian Journal of Fisheries and Aquatic Sciences, 72, 1–12.

[ece37884-bib-0118] Moser, D., Roesti, M., & Berner, D. (2012). Repeated lake‐stream divergence in stickleback life history within a central European lake basin. Public Library of Science One, 7, e50620. 23226528 10.1371/journal.pone.0050620 PMC3514289

[ece37884-bib-0119] Narum, S. R., Zendt, J. S., Graves, D., & Sharp, W. R. (2008). Influence of landscape on resident and anadromous life history types of *Oncorhynchus mykiss* . Canadian Journal of Fishereis and Aquatic Sciences, 65, 1013–1023.

[ece37884-bib-0120] Neave, F. B., Steeves, T. B., Pratt, T. C., McLaughlin, R. L., Adams, J. V., & Docker, M. F. (2019). Stream characteristics associated with feeding type in silver (*Ichthyomyzon unicuspis*) and northern brook (*I. fossor*) lampreys and tests for phenotypic plasticity. Environmental Biology of Fishes, 102, 615–627.

[ece37884-bib-0121] Nichols, K. M., Kozfkay, C. C., & Narum, S. R. (2016). Genomic signatures among *Oncorhynchus nerka* ecotypes to inform conservation and management of endangered sockeye salmon. Evolutionary Applications, 9, 1285–1300. 27877206 10.1111/eva.12412 PMC5108219

[ece37884-bib-0122] Nielsen, J. L., & Fountain, M. C. (1997). Microsatellite diversity in sympatric reproductive ecotypes of Pacific steelhead (*Oncorhynchus mykiss*) from the Middle Fork Eel River, California. Ecology of Freshwater Fish, 8, 159–168.

[ece37884-bib-0123] Ohms, H. A., Sloat, M. R., Reeves, G. H., Jordan, C. E., & Dunham, J. B. (2014). Influence of sex, migration distance, and latitude on life history expression in steelhead and rainbow trout (*Oncorhynchus mykiss*). Canadian Journal of Fisheries and Aquatic Sciences, 71, 70–80.

[ece37884-bib-0124] Oravec, T. J., & Reimchen, T. E. (2013). Divergent reproductive life histories in Haida Gwaii stickleback (*Gasterosteus* spp.). Canadian Journal of Zoology, 91, 17–24.

[ece37884-bib-0125] Orlov, A., & Beamish, R. (2016a). Jawless fishes of the world, Vol. 1. Cambridge Scholars Publishing.

[ece37884-bib-0126] Orlov, A., & Beamish, R. (2016b). Jawless fishes of the world, Vol. 2. Cambridge Scholars Publishing.

[ece37884-bib-0127] Palacios, M. G., Sparkman, A. M., & Bronikowski, A. M. (2012). Corticosterone and pace of life in two life‐history ecotypes of the garter snake *Thamnophis elegans* . General and Comparative Endocrinology, 175, 443–448. 10.1016/j.ygcen.2011.11.042 22178432

[ece37884-bib-0128] Parker, K., Hess, J. E., Narum, S. R., & Kinziger, A. P. (2019). Evidence for the genetic basis and epistatic interactions underlying ocean‐ and river‐maturing ecotypes of Pacific Lamprey (*Entosphenus tridentatus*) returning to the Klamath River, California. Molecular Ecology, 28, 3171–3185. 31132186 10.1111/mec.15136

[ece37884-bib-0129] Patimar, R., Najafabadi, M. H., & Souraki, M. G. (2010). Life history features of the nonindigenous three spined stickleback (*Gasterosteus aculeatus* Linnaeus, 1758) in the Gomishan wetland (southeast Caspian Sea, Iran). Turkish Journal of Zoology, 34, 461–470.

[ece37884-bib-0130] Patten, M. A. (2015). Subspecies and the philosophy of science. The Auk, 132, 481–485. 10.1642/AUK-15-1.1

[ece37884-bib-0131] Pavey, S. A., Hamon, T. R., & Nielsen, J. L. (2007). Revisiting evolutionary dead ends in sockeye salmon (*Oncorhynchus nerka*) life history. Canadian Journal of Fisheries and Aquatic Sciences, 64, 1199–1208.

[ece37884-bib-0132] Penaluna, B. E., Abadía‐Cardoso, A., Dunham, J. B., García‐Dé León, F. J., Gresswell, R. E., Luna, A. R., Taylor, E. B., Shepard, B. B., Al‐Chokhachy, R., Muhlfeld, C. C., Bestgen, K. R., Rogers, K., Escalante, M. A., Keeley, E. R., Temple, G. M., Williams, J. E., Matthews, K. R., Pierce, R., Mayden, R. L., … Fausch, K. D. (2016). Conservation of native Pacific trout diversity in western North America. Fisheries, 41, 286–300. 10.1080/03632415.2016.1175888

[ece37884-bib-0133] Phillis, C. C., Moore, J. W., Buoro, M., Hayes, S. A., Garza, J. C., & Pearse, D. E. (2016). Shifting thresholds: Rapid evolution of migratory life histories in steelhead/rainbow trout, *Oncorhynchus mykiss* . Journal of Hereditary, 107, 51–60. 10.1093/jhered/esv085 26585381

[ece37884-bib-0134] Poizat, G., Rosecchi, E., & Crivelli, A. J. (2002). Life‐history variation within a three‐spined stickleback population in the Camargue. Journal of Fish Biology, 60, 1296–1307. 10.1111/j.1095-8649.2002.tb01721.x

[ece37884-bib-0135] Potter, I. C., Gill, H. S., Renaud, C. B., & Haoucher, D. (2015). The taxonomy, phylogeny, and distribution of lampreys. In M. F. Docker (Ed.), Lampreys: Biology, conservation and control (Vol. 1, pp. 35–74). Fish and Fisheries Monograph Series,Springer.

[ece37884-bib-0136] Proćków, M., Proćków, J., Błażej, P., & Mackiewicz, P. (2018). The influence of habitat preferences on shell morphology in ecophenotypes of *Trochulus hispidus* complex. Science of the Total Environment, 630, 1036–1043. 10.1016/j.scitotenv.2018.02.311 29554725

[ece37884-bib-0137] Quinn, T. P. (2005). The behavior and ecology of Pacific salmon and trout. University of Washington Press.

[ece37884-bib-0138] Quinn, T. P., Hendry, A. P., & Wetzel, L. A. (1995). The influence of life history trade‐offs and the size of incubation gravels on egg size variation in sockeye salmon (*Oncorhynchus nerka*). Oikos, 74, 425–438. 10.2307/3545987

[ece37884-bib-0139] Quinn, T. P., & Myers, K. W. (2004). Anadromy and the marine migrations of Pacific salmon and trout: Rounsefell revisited. Reviews in Fish Biology and Fisheries, 14, 421–442. 10.1007/s11160-005-0802-5

[ece37884-bib-0140] Quinn, T. P., & Unwin, M. J. (1993). Variation in life history patterns among New Zealand Chinook salmon (*Oncorhynchus tshawytscha*) populations. Canadian Journal of Fisheries and Aquatic Sciences, 50, 1414–1421.

[ece37884-bib-0141] Raeymaekers, J. A. M., Boisjoly, M., Delaire, L., Berner, D., Räsänen, K., & Hendry, A. P. (2010). Testing for mating isolation between ecotypes: Laboratory experiments with lake, stream and hybrid stickleback. Journal of Evolutionary Biology, 23, 2694–2708. 20939859 10.1111/j.1420-9101.2010.02133.x

[ece37884-bib-0142] Rasmussen, J. B., Robinson, M. D., & Heath, D. D. (2010). Ecological consequences of hybridization between natie westslope cutthroat (*Oncorhynchus clarkii lewisi*) and introduced rainbow (*Oncorhynchus mykiss*) trout: Effects on life history and habitat use. Canadian Journal of Fisheries and Aquatic Sciences, 67, 357–370.

[ece37884-bib-0143] Rastorguev, S. M., Nedoluzhko, A. V., Gruzdeva, N. M., Boulygina, E. S., Tsygankova, S. V., Oshchepkov, D. Y., Mazur, A. M., Prokhortchouk, E. B., & Skryabin, K. G. (2018). Gene expression in the three‐spined stickleback (*Gasterosteus aculeatus*) of marine and freshwater ecotypes. Acta Naturae, 10, 66–74. 10.32607/20758251-2018-10-1-66-74 29713520 PMC5916735

[ece37884-bib-0144] Rastorguev, S. M., Nedoluzhko, A. V., Sharko, F. S., Boulygina, E. S., Sokolov, A. S., Gruzdeva, N. M., Skryabin, K. G., & Prokhortchouk, E. B. (2016). Identificiation of novel micro RNA genes in freshwater and marine ecotypes of the three‐spined stickleback (*Gasterosteus aculeatus*). Molecular Ecology Resources, 16, 1491–1498. 27238497 10.1111/1755-0998.12545

[ece37884-bib-0145] Rich, H. B. Jr, Quinn, T. P., Scheuerell, M. D., & Schindler, D. E. (2009). Climate and intraspecific competition control the growth and life history of juvenile sockeye salmon (*Oncorhynchus nerka*) in Iliama Lake, Alaska. Canadian Journal of Fisheries and Aquatic Sciences, 66, 238–246.

[ece37884-bib-0146] Riva‐Rossi, C., Barrasso, D., Baker, C., Quiroga, A. P., Baigun, C., & Basso, N. (2020). Revalidation of the Argentinian pouched lamprey *Geotria macrostoma* (Burmeister, 1868) with Molecular and Morphological Evidence. Public Library of Science One, 15, e0233792. 10.1371/journal.pone.0233792 32470001 PMC7259705

[ece37884-bib-0147] Roddam, M., & Ward, D. M. (2015). Life‐history differencces of juvenile Chinook salmon *Oncorhynchus tshawytscha* across rearing locations in the Shasta River, California. Ecology of Freshwater Fish, 26, 150–159.

[ece37884-bib-0148] Rollins, J. L., Chiang, P., Waite, J. N., Von Hippel, F. A. , & Bell, M. A. (2017). Jacks and jills: Alternative life history phenotypes and skewed sex ratio in anadromous threespine stickleback (*Gasterosteus aculeatus*). Evolutionary Ecology Research, 18, 363–382.

[ece37884-bib-0149] Rougemont, Q., Gagnaire, P.‐A., Perrier, C., Genthon, C., Besnard, A.‐L., Launey, S., & Evanno, G. (2017). Inferring the demographic history underlying parallel genomic divergence among pairs of parasitic and nonparasitic lamprey ecotypes. Molecular Ecology, 26, 142–162. 27105132 10.1111/mec.13664

[ece37884-bib-0150] Rougemont, Q., Gaigher, A., Lasne, E., Côte, J., Coke, M., Besnard, A.‐L., Launey, S., & Evanno, G. (2015). Low reproductive isolation and highly variable levels of gene flow reveal limited progress towards speciation between European river and brook lampreys. Journal of Evolutionary Biology, 28, 2248–2263. 10.1111/jeb.12750 26348652

[ece37884-bib-0151] Saito, S., Shimizu, I., Seki, J., & Nagasawa, K. (2009). Relationships between zooplankton abundance and the early marine life history of juvenile chum salmon *Oncorhynchus keta* in eastern Hokkaido, Japan. Fisheries Science, 75, 303–316.

[ece37884-bib-0152] Saito, T., & Nakano, S. (1999). Reproductive timing‐dependent alternation of offspring life histories in female threespine sticklebacks. Canadian Journal of Zoology, 77, 1314–1321. 10.1139/z99-104

[ece37884-bib-0153] Salewski, V. (2003). Satellite species in lampreys: A worldwide trend for ecological speciation in sympatry? Journal of Fish Biology, 63, 267–279. 10.1046/j.1095-8649.2003.00166.x

[ece37884-bib-0154] Samarasin, P., Shuter, B. J., & Rodd, F. H. (2017). After 100 years: Hydroelectric dam‐induced life history divergence and population genetic changes in sockeye salmon (*Oncorhynchus nerka*). Conservation Genetics, 18, 1449–1462.

[ece37884-bib-0155] Schade, F. M., Clemmesen, C., & Wegner, K. M. (2014). Within‐ and transgenerational effects of ocean acidification on life history of marine three‐spined stickleback (*Gasterosteus aculeatus*). Marine Biology, 161, 1667–1676. 10.1007/s00227-014-2450-6

[ece37884-bib-0156] Scharsack, J. P., & Kalbe, M. (2014). Differences in susceptibility and immune responses of three‐spined stickleback from lake and river ecotypes to sequential infections with the eye fluke *Diplostomum pseudospathaceum* . Parasites and Vectors, 7, 109–119. 24656136 10.1186/1756-3305-7-109 PMC3994412

[ece37884-bib-0157] Scharsack, J. P., Kalbe, M., Harrod, C., & Rauch, G. (2007). Habitat‐specific adaptation of immune responses of stickleback (*Gasterosteus aculeatus*) lake and river ecotypes. Proceedings of the Royal Society B, 274, 1523–1532. 17426014 10.1098/rspb.2007.0210 PMC2176159

[ece37884-bib-0158] Schluter, D. (2010). Resource competition and coevolution in sticklebacks. Evolution: Education and Outreach, 3, 54–61. 10.1007/s12052-009-0204-6

[ece37884-bib-0159] Schluter, D., & Conte, G. L. (2009). Genetics and ecological speciation. Proceedings of the National Academy of Sciences of the United States of America, 106, 9955–9962. 10.1073/pnas.0901264106 19528639 PMC2702799

[ece37884-bib-0160] Schönborn, W., & Peschke, T. (1988). Biometric studies on species, races, ecophenotypes and individual variations of soil‐inhabiting Testacea (Protozoa, Rhizopoda), including *Trigonopyxis minuta* nsp. and *Corythion asperulum* nsp. Archiv für Protistenkunde, 136, 345–363.

[ece37884-bib-0161] Segura‐Trujillo, C. A., Willig, M. R., & Álvarez‐Castañeda, S. T. (2018). Correspondence between ecomorphotype and use of arthropod resources by bats of the genus *Myotis* . Journal of Mammalogy, 99, 659–667.

[ece37884-bib-0162] Sharma, R., & Quinn, T. P. (2012). Linkages between life history type and migration pathways in freshwater and marine environments for Chinook salmon, *Oncorhynchus tshawytscha* . Oecologia, 41, 1–13. 10.1016/j.actao.2012.03.002

[ece37884-bib-0163] Skúlason, S., Parsons, K. J., Richard Svanbäck, K., Räsänen, M. M., Ferguson, C. E., Adams, P.‐A., Amundsen, P., Bartels, C. W., Bean, J. W., Boughman, G., Englund, J., Guðbrandsson, O. E., Hooker, A. G., Hudson, K. K., Kahilainen, R., Knudsen, B. K., Kristjánsson, C.‐ A.‐L., Leblanc, Z., Jónsson, G., … Snorrason, S. S. (2019). A way forward with eco evo devo: An extended theory of resource polymorphism with postglacial fishes as model systems. Biological Reviews, 94, 1786–1808. 31215138 10.1111/brv.12534 PMC6852119

[ece37884-bib-0164] Skúlason, S., & Smith, T. B. (1995). Resource polymorphisms in vertebrates. Trends in Ecology and Evolution, 10, 366–370. 21237070 10.1016/s0169-5347(00)89135-1

[ece37884-bib-0165] Sloat, M. R., & Reeves, G. H. (2014). Individual condition, standard metabolic rate, and rearing temperature influence steelhad and rainbow trout (*Oncorhynchus mykiss*) life histories. Canadian Journal of Fisheries and Aquatic Sciences, 71, 491–501.

[ece37884-bib-0166] Snover, M. L., Watters, G. M., & Mangel, M. (2006). Top‐down and bottom‐up control of life history strategies in coho salmon (*Oncorhynchus kisutch*). The American Naturalist, 167, E140–E157. 10.1086/502804 16671006

[ece37884-bib-0167] Snyder, R. J. (1991). Migration and life histories of the threespine stickleback: Evidence for adaptive variation in growth rate between populations. Environmental Biology of Fishes, 31, 381–388. 10.1007/BF00002363

[ece37884-bib-0168] Snyder, R. J., & Dingle, H. (1990). Effects of fresh water and marine overwintering environments on life histories of threespine sticklebacks: Evidence for adaptive variation between anadromous and resident freshwater populations. Oecologia, 84, 386–390. 10.1007/BF00329764 28313030

[ece37884-bib-0169] Sogard, S. M., Merz, J. E., Satterthwaite, W. H., Beakes, M. P., Swank, D. R., Collins, E. M., Titus, R. G., & Mangel, M. (2012). Contrasts in habitat characteristics and life history patterns of *Oncorhynchus mykiss* in California Central Coast and Central Valley. Transactions of the American Fisheries Society, 141, 747–760.

[ece37884-bib-0170] Sorensen, F. E., & Lindberg, D. R. (1991). Preferential predation by American black oystercatchers on transitional ecophenotypes of the limpet *Lottia pelta* (Rathke). Journal of Experimental Marine Biology and Ecology, 154, 123–136. 10.1016/0022-0981(91)90078-B

[ece37884-bib-0171] Spice, E. K., Goodman, D. H., Reid, S. B., & Docker, M. F. (2012). Neither philopatric nor panmictic: Microsatellite and mtDNA evidence suggests lack of natal homing but limits to dispersal in Pacific lamprey. Molecular Ecology, 21, 2916–2930. 10.1111/j.1365-294X.2012.05585.x 22564149

[ece37884-bib-0172] Spice, E. K., Whitesel, T. A., Silver, G. S., & Docker, M. F. (2019). Contemporary and historical river connectivity influence population structure in western brook lamprey in the Columbia Basin. Conservation Genetics, 20, 299–314.

[ece37884-bib-0174] Stearley and Smith . (1993). Phylogeny of the Pacific trouts and salmon (*Oncorhynchus*) and genera of the family salmonidae. Transactions of the American Fisheries Society, 122, 1–33.

[ece37884-bib-0175] Stearns, S. C. (1989). Trade‐offs in life‐history evolution. Functional Ecology, 3, 259–268. 10.2307/2389364

[ece37884-bib-0176] Stearns, S. C. (1992). The evolution of life histories. Oxford University Press Inc.

[ece37884-bib-0177] Stearns, S. C., & Hendry, A. P. (2004). Introduction: The salmonid contribution to key issues in evolution. In A. P. Hendry, & S. C. Stearns (Eds.), Evolution illuminated: Salmon and their relatives (pp. 3–19). Oxford University Press.

[ece37884-bib-0178] Tallman, R. F., & Healey, M. C. (1991). Phenotypic differentiation in seasonal ecotypes of chum salmon, *Oncorhynchus keta* . Canadian Journal of Fisheries and Aquatic Sciences, 48, 661–671.

[ece37884-bib-0179] Taylor, E. B. (1990a). Variability in agnostic behaviour and salinity tolerance between and within two populations of juvenile Chinook salmon, *Oncorhynchus tshawytscha*, with contrasting life histories. Journal of Fisheries and Aquatic Sciences, 47, 2172–2180.

[ece37884-bib-0180] Taylor, E. B. (1990b). Environmental correlates of life‐history variation in juvenile Chinook salmon, *Oncorhynchus tshawytscha* (Walbaum). Journal of Fish Biology, 37, 1–17.

[ece37884-bib-0181] Taylor, E. B. (1990c). Phenotypic correlates of life‐history variation in juvenile Chinook salmon, *Oncorhynchus tshawytscha* . Journal of Animal Ecology, 59, 455–468.

[ece37884-bib-0182] Taylor, E. B. (1999). Species pairs of north temperate freshwater fishes: Evolution, taxonomy, and conservation. Reviews in Fish Biology and Fisheries, 9, 299–324.

[ece37884-bib-0183] Taylor, E. B., Foote, C. J., & Wood, C. C. (1996). Molecular genetic evidence for parallel life‐history evolution within a Pacific salmon (sockeye slamon and kokanee, *Oncorhynchus nerka*). Evolution, 50, 401–416. 28568856 10.1111/j.1558-5646.1996.tb04502.x

[ece37884-bib-0184] Taylor, E. B., Harvey, S., Pollard, S., & Volpe, J. (1997). Postglacial genetic differentiation of reproductive ecotypes of kokanee *Oncorhynchus nerka* in Okanagan Lake, British Columbia. Molecular Ecology, 6, 503–517. 9200826 10.1046/j.1365-294x.1997.00213.x

[ece37884-bib-0185] Thériault, V., Moyer, G. R., & Banks, M. A. (2010). Survival and life history characteristics among wild and hatchery coho salmon (*Oncorhynchus kisutch*) returns: How do unfed fry differ from smolt releases? Canadian Journal of Fisheries and Aquatic Sciences, 67, 486–497.

[ece37884-bib-0186] Thorpe, J. E., Mangel, M., Metcalfe, N. B., & Huntingford, F. A. (1998). Modelling the proximate basis of salmonid life history variation, with application to Atlantic salmon, *Salmo salar* L. Evolutionary Ecology, 12, 581–599. 10.1023/A:1022351814644

[ece37884-bib-0187] Tsiger, V. V., Skirin, V. I., Krupyanko, N. I., Kashkin, K. A., & Semenchenko, A. Y. (1994). Life history forms of male masu salmon (*Oncorhynchus masou*) in South Primoré, Russia. Canadian Journal of Fisheries and Aquatic Sciences, 51, 197–208.

[ece37884-bib-0188] Turesson, G. (1922). The species and the variety as ecological unit. Hereditas, 3, 100–113.

[ece37884-bib-0189] Unwin, M. J., & Glova, G. J. (1997). Changes in life history parameters in a naturally spawning population of Chinook salmon…. Associated with releases of hatchery‐reared fish. Canadian Journal of Fisheries and Aquatic Sciences, 54, 1235–1245.

[ece37884-bib-0191] Van Doornik, D. M., Berejikian, B. A., & Campbell, L. A. (2013). Gene flow between sympatric life history forms of *Oncorhynchus mykiss* located above and below migratory barriers. Public Library of Science One, 8, 379931. 10.1371/journal.pone.0079931 PMC3818241 24224023

[ece37884-bib-0192] Van Doornik, D. M., Berejikian, B. A., Campbell, L. A., & Volk, E. C. (2010). The effect of a supplementation program on the genetic and life history characteristics of an *Oncorhynchus mykiss* population. Canadian Journal of Fisheries and Aquatic Sciences, 67, 1449–1458. 10.1139/F10-073

[ece37884-bib-0193] Vladykov, V. D., & Kott, E. (1979). Satellite species among the holarctic lampreys (Petromyzontidae). Canadian Journal of Zoology, 57, 860–867.

[ece37884-bib-0194] Waples, R. S. (1991). Pacific Salmon, *Oncorhynchus* spp., and the definition of "Species" Under the Endangered Species Act. Marine Fisheries Review, 53, 11–22.

[ece37884-bib-0195] Waples, R. S. (1995). Evolutionary Significant Units and the conservation of biological diversity under the Endangered Species Act. American Fisheries Society Symposium, 17, 8–27.

[ece37884-bib-0196] Waples, R. S. (2006). Distinct Population Segments. In J. M. Scott, D. D. Goble, & F. W. Davis (Eds.), The Endangered Species Act at thirty, volume 2: Conserving biodiversity in human‐dominated landscapes (pp. 127–149). Island Press.

[ece37884-bib-0197] Waples, R. S., Aebersold, P. B., & Winans, G. A. (2011). Population genetic structure and life history variability in *Oncorhynchus nerka* from the Snake River Basin. Transactions of the American Fisheries Society, 140, 716–733.

[ece37884-bib-0198] Waples, R. S., Gustafson, R. G., Weitkamp, L. A., Myers, J. M., Johnson, O. W., Busby, P. J., Hard, J. J., Bryant, G. J., Waknitz, F. W., Neely, K., Teel, D., Grant, W. S., Winans, G. A., Phelps, S., Marshall, A., & Baker, B. M. (2001). Characterizing diversity in salmon from the Pacific Northwest. Journal of Fish Biology, 59(Suppl. A), 1–41. 10.1111/j.1095-8649.2001.tb01376.x

[ece37884-bib-0199] Waples, R. S., & Hendry, A. P. (2008). Special issue: Evolutionary perspectives on salmonid conservation and management. Evolutionary Applications, 1, 183–188. 10.1111/j.1752-4571.2008.00035.x 25567625 PMC3352439

[ece37884-bib-0200] Waples, R. S., Pess, G. R., & Beechie, T. M. (2008). Evolutionary history of Pacific salmon in dynamic environments. Evolutionary Applications, 1, 189–206. 10.1111/j.1752-4571.2008.00023.x 25567626 PMC3352440

[ece37884-bib-0202] Westley, P. A. H., Quinn, T. P., & Dittman, A. H. (2013). Rates of straying by hatchery‐produced Pacific salmon (*Oncorhynchus* spp.) and steelhead (*O. mykiss*) differ among species, life history types, and populations. Canadian Journal of Fisheries and Aquatic Sciences, 70, 735–746.

[ece37884-bib-0203] Willacker, J. J., von Hippel, F. A., Ackerly, K. L., & O’Hara, T. M. (2013). Habitat‐specific foraging and sex determine mercury concentrations in sympatric benthic and limnetic ecotypes of threespine stickleback. Environmental Toxicology and Chemistry, 32, 1623–1630. 10.1002/etc.2213 23456641 PMC3684275

[ece37884-bib-0204] Winemiller, K. O. (2005). Life history strategies, population regulation, and implications for fisheries management. Canadian Journal of Fisheries and Aquatic Sciences, 62, 872–885.

[ece37884-bib-0205] Winemiller, K. O., & Rose, K. A. (1992). Patterns of life‐history diversification in North American fishes: Implications for population regulation. Canadian Journal of Fisheries and Aquatic Sciences, 49, 2196–2218. 10.1139/f92-242

[ece37884-bib-0206] Wood, C. C., Bickham, J. W., Nelson, R. J., Foote, C. J., & Patton, J. C. (2008). Recurrent evolution of life history ecotypes in sockeye salmon: Implications for conservation and future evolution. Evolutionary Applications, 1, 207–221. 10.1111/j.1752-4571.2008.00028.x 25567627 PMC3352436

[ece37884-bib-0207] Wootton, R. J. (2009). The Darwinian stickleback *Gasterosteus aculeatus*: A history of evolutionary studies. Journal of Fish Biology, 75, 1919–1942. 10.1111/j.1095-8649.2009.02412.x 20738666

[ece37884-bib-0208] Wund, M. A., Valena, S., Wood, S., & Baker, J. A. (2012). Ancestral plasticity and allometry in threespine stickleback reveal phenotypes associated with derived, freshwater ecotypes. Biological Journal of the Linnean Society, 105, 573–583. 22611287 10.5061/dryad.hb824gd4 PMC3351840

[ece37884-bib-0209] Yamamoto, T. (2004). Sex‐specific growth pattern during early life history in masu salmon, *Oncorhynchus masou* . Ecology of Freshwater Fish, 13, 203–207.

[ece37884-bib-0210] Yamamoto, T., & Edo, K. (2002). Reproductive behaviors related to life history forms in male masu salmon, *Oncorhynchus masou* Breboort, in Lake Toya, Japan. Journal of Freshwater Ecology, 17, 275–281.

[ece37884-bib-0211] Yamazaki, Y., & Nagai, T. (2013). Directional selection against different life histories in the Arctic lamprey (*Lethenteron camtschaticum*): Identification by microsatellite analysis. Canadian Journal of Fisheries and Aquatic Sciences, 70, 825–829.

[ece37884-bib-0212] Youson, J. H., & Beamish, R. J. (1991). Comparison of the internal morphology of adults of a population of lampreys that contains a nonparasitic life‐history type, *Lampetra richardsoni*, and a potentially parasitic form, *L. richardsoni* var. *marifuga* . Canadian Journal of Zoology, 69, 628–637.

[ece37884-bib-0213] Youson, J. H., Heinig, J. A., Khanam, S. F., Sower, S. A., Kawauchi, H., & Keeley, F. W. (2006). Patterns of proopiomelanotropin and proopiocortin gene expression and of immunochemistry for gonadotropin‐releasing hormones (lGnRH‐I and III) during the life cycle of a nonparasitic lamprey: Relationship to this adult life history type. General and Comparative Endocrinology, 148, 54–71. 16364323 10.1016/j.ygcen.2005.10.015

[ece37884-bib-0214] Zazzo, A., Smith, G. R., Patterson, W. P., & Dufour, E. (2006). Life history reconstruction of modern and fossil sockeye salmon….by oxygen isotope analysis of otoliths, vertebrae, and teeth: Implication for paleoenvironmental reconstructions. Earth and Planetary Science Letters, 249, 200–215.

[ece37884-bib-0215] Zerrenner, A., & Marsden, J. E. (2005). Influence of larval sea lamprey density on transformer life history characteristics in Lewis Creek, Vermont. Transactions of the American Fisheries Society, 134, 687–696. 10.1577/T04-015.1

[ece37884-bib-0216] Zerrenner, A., & Marsden, J. E. (2006). Comparison of larval sea lamprey life history characteristics in a lampricide‐treated tributary and untreated tributary system of Lake Champlain. Transactions of the American Fisheries Society, 135, 1301–1311. 10.1577/T05-012.1

